# Restriction of *Wolbachia* Bacteria in Early Embryogenesis of Neotropical *Drosophila* Species via Endoplasmic Reticulum-Mediated Autophagy

**DOI:** 10.1128/mbio.03863-21

**Published:** 2022-03-31

**Authors:** Anton Strunov, Katy Schmidt, Martin Kapun, Wolfgang J. Miller

**Affiliations:** a Center for Anatomy and Cell Biology, Department of Cell and Developmental Biology, Medical University of Vienna, Vienna, Austria; b Central Research Laboratories, Natural History Museum Vienna, Vienna, Austria; EPFL

**Keywords:** symbiosis, *Drosophila*, *Wolbachia*, tropism, autophagy, development, germline, stem cell, tropism control

## Abstract

*Wolbachia* are maternally transmitted intracellular bacteria that are not only restricted to the reproductive organs but also found in various somatic tissues of their native hosts. The abundance of the endosymbiont in the soma, usually a dead end for vertically transmitted bacteria, causes a multitude of effects on life history traits of their hosts, which are still not well understood. Thus, deciphering the host-symbiont interactions on a cellular level throughout a host’s life cycle is of great importance to understand their homeostatic nature, persistence, and spreading success. Using fluorescent and transmission electron microscopy, we conducted a comprehensive analysis of *Wolbachia* tropism in soma and germ line of six *Drosophila* species at the intracellular level during host development. Our data uncovered diagnostic patterns of infections to embryonic primordial germ cells and to particular cells of the soma in three different neotropical *Drosophila* species that have apparently evolved independently. We further found that restricted patterns of *Wolbachia* tropism are determined in early embryogenesis via selective autophagy, and their spatially restricted infection patterns are preserved in adult flies. We observed tight interactions of *Wolbachia* with membranes of the endoplasmic reticulum, which might play a scaffolding role for autophagosome formation and subsequent elimination of the endosymbiont. Finally, by analyzing *D. simulans* lines transinfected with nonnative *Wolbachia*, we uncovered that the host genetic background regulates tissue tropism of infection. Our data demonstrate a novel and peculiar mechanism to limit and spatially restrict bacterial infection in the soma during a very early stage of host development.

## INTRODUCTION

*Wolbachia* are endosymbiotic bacteria residing within cells of many arthropod and nematode species (reviewed in reference 1). Most of these host-microbe associations are considered facultative and even pathogenic (2), although cases of obligate mutualism also exist (3–7). In insects, high transgenerational infectivity and maintenance of *Wolbachia* is ensured by its successful transovarial transmission (reviewed in references 8 and 9), although horizontal transmission also occurs (reviewed in references 10 and 11). Thus, the microbe mostly relies on colonization of the female germ line to be stably transmitted to the next generation (1, 12). However, the infection is not solely confined to reproductive organs and can be found in different somatic tissues, like the central nervous system (CNS), retina, fat body, muscles, hemolymph, and Malpighian tubules of a host (reviewed in reference 10). Such a variety of bacterial localization brings about a wide range of effects on host fitness and behavior (reviewed in reference 13). Moreover, regulation of *Wolbachia* density within somatic tissues is a key factor in host-symbiont association, strongly affecting both host survival and persistence of bacteria in a population (2, 14–16). The rich somatic life of the bacteria provides a scarcely studied repertoire of intimate cell-specific interactions balancing host-microbe association. Understanding its essence is of great importance for fundamental knowledge as well as for application in biological control of invertebrate pests and vectors of diseases (reviewed in reference 17).

The neotropical *Drosophila* species *D. paulistorum*, *D. willistoni*, and *D. tropicalis* (willistoni group) as well as *D. septentriosaltans* and *D. sturtevanti* (saltans group) represent unique models for studying host-microbe interactions due to their long-term history of coevolution with *Wolbachia* endosymbionts (6, 18). Each of these neotropical *Drosophila* species carries a specific *Wolbachia* strain, which exhibits either obligate mutualistic (*D. paulistorum*) or facultative (the other four host species) relationships. Among these neotropical *Wolbachia* strains, *w*Pau, *w*Wil, *w*Tro, and *w*Spt from *D. paulistorum*, *D. willistoni*, *D. tropicalis*, and *D. septentriosaltans* are closely related to each other and belong to the *w*Au-like group, whereas *w*Stv from *D. sturtevanti* is the most distantly related to the rest (15, 18). All strains used in our present study represent high-titer *Wolbachia* infections, which are easily detected with standard PCR (6, 18) and do not require additional low-titer detection methods (19). In embryos of *D. willistoni* and *D. paulistorum*, native *Wolbachia* are mainly restricted to the primordial germ cells (PGCs), the future germ line, whereas palearctic fly hosts like D. melanogaster and *D. simulans* embryos show systemic infections with no defined tropism (6, 18).

We have furthermore uncovered the spatial and asymmetric restriction of *Wolbachia* in *D. paulistorum* to defined larval and adult brain regions (20), which might be linked to the symbiont-directed assortative mating behavior observed in this obligate host-microbe association (6, 7). However, it remains unclear (i) if the PGC and neural restrictions are unique to *D. paulistorum* hosts, (ii) at which developmental stages the tropism is established, and (iii) by which cellular mechanism(s) the germ line and somatic *Wolbachia* restrictions are achieved. Such diverse types of host-microbe interactions provide an opportunity to decipher the mechanistic basis for their tropism to defined somatic and germ line tissues as well as their density within a cell.

By using fluorescent *in situ* hybridization (FISH) with *Wolbachia*-specific probes throughout host development, we uncovered spatial and temporal dynamics of both the “systemic” and “restricted” infection types in six native *Drosophila* hosts. With the help of sequential *Wolbachia*-FISH and immunofluorescence, we showed that the distribution of infection is determined already during early embryogenesis with elimination of *Wolbachia* from most of the embryonic cells, but not PGCs, through autophagy. This is followed by the spatial restriction of *Wolbachia* to the future gonads and a few particular areas of somatic tissues in the adult. With the help of transmission electron microscopy, we mapped out the early stages of the bacterial elimination process and could demonstrate that the endoplasmic reticulum (ER) tightly encircling *Wolbachia* in early-cellularized blastodermal embryos might serve as a scaffold for assembly of the autophagy machinery. Finally, by transferring a natively restricted *Wolbachia* strain into a systemic background, we decipher that the host background plays a major role in regulating the infection tropism in tissues.

## RESULTS

### *Wolbachia* infection is restricted to specific areas of the soma and the germ line of some neotropical *Drosophila* species.

In an earlier publication we showed that, contrary to the systemic infections in D. melanogaster and *D. simulans* (21), *Wolbachia* of neotropical *D. paulistorum* flies are tightly restricted to certain brain areas (20). In the present study, we investigated whether such an explicit isolation of infection in the nervous tissue is an exceptional case for *D. paulistorum* flies or similar examples of bacterial restriction could be found in other related host species. We analyzed the distribution of native *Wolbachia* in both soma and germ line of five other neotropical *Drosophila* species (*D. paulistorum*, *D. willistoni*, *D. tropicalis*, *D. septentriosaltans*, and *D. sturtevanti*), with D. melanogaster as a representative for the systemic infection (20). Finally, we tested bacterial tropism in a *de novo* host-symbiont association by transinfecting the systemic host *D. simulans* (STC) with the *Wolbachia* strain *w*Wil from *D. willistoni*, a representative of the restriction type we named *w*Wil/STC (Table 1). For the sake of simplicity in the following text, we use SIT and RIT abbreviations to define systemic infection type and restricted infection type, respectively.

**TABLE 1:** *Drosophila* species and lines used in the study

*Drosophila* species	Subgroup	Line code	Short name	*Wolbachia* strain
D. melanogaster	Melanogaster	Harwich H2	MEL	*w*Mel
*D. simulans*	Melanogaster	KB30STC	STC	*w*Au
*D. tropicalis*	Willistoni	Trop1	TRO	*w*Tro
*D. paulistorum*	Willistoni	Pau5 O11	PAU	*w*Pau
*D. willistoni*	Willistoni	JS6.3	WIL	*w*Wil
*D. septentriosaltans*	Saltans	SEP1/PLR	SPT	*w*Spt
*D. prosaltans*	Saltans	Pro1	PRO	*w*Pro
*D. sturtevanti*	Sturtevanti	FG707	STV	*w*Stv
*D. lehrmanae*	Sturtevanti	FG583	LEH	*w*Leh
*D. simulans* TI^*a*^	Melanogaster	wilE/STC 36	wilE/STC	*w*Wil

a Transinfected by microinjection.

### Tropism of *Wolbachia* in adult and larval nervous tissues of *Drosophila*.

**FIG 1:**
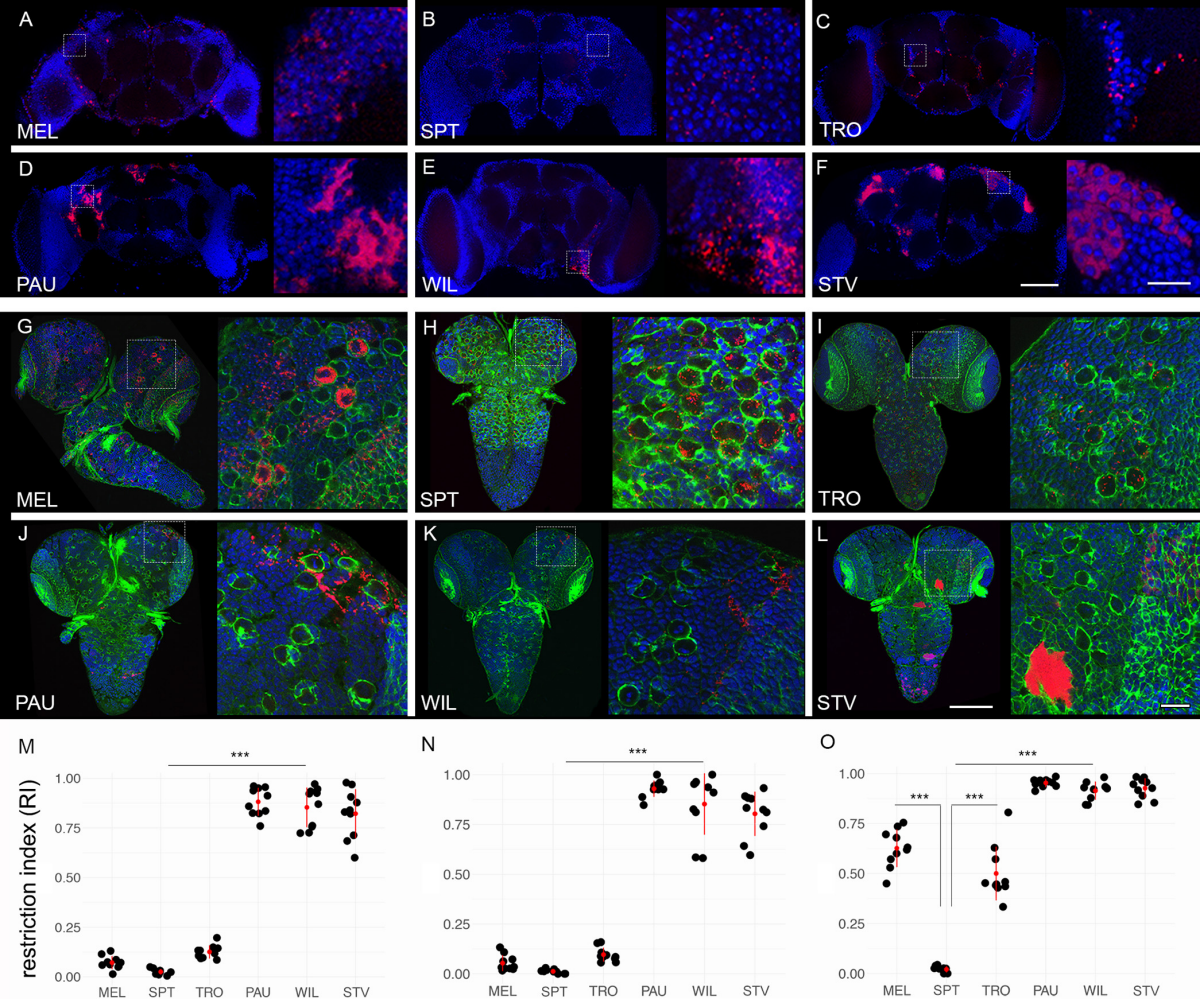
Restriction of *Wolbachia* infection in nervous tissues of neotropical *Drosophila*. Fluorescent *in situ* hybridization on different *Drosophila* adult brains (A to F) and 3rd-instar larval CNS (G to L) using 16S rRNA *Wolbachia*-specific probe (red). The bottom plots show restriction indices of all six species for *Wolbachia* infections in adult brains (N) and larval CNS (M), respectively. O shows RI of bacterial infection in neuroblasts of 3rd-instar larval CNS. DNA is stained with DAPI (blue) and actin with phalloidin (green). For each *Drosophila* species 10 organs from each developmental stage were analyzed (see Data Set S1). Asterisks denote statistical significance (***, *P < *0.001; Poisson regression). Red bars show standard deviations, red dots designate the mean value. Scale bar, 50 μm.

We conducted fluorescent *in situ* hybridization (FISH) analysis using *Wolbachia*-specific 16S rRNA probes to survey the bacterial distribution in adult brains of all six native host species listed above. As shown in Fig. 1A and C, *D. septentriosaltans* (SPT) and *D. tropicalis* (TRO) exhibit, similar to D. melanogaster (MEL), a SIT pattern with bacteria evenly distributed all over the tissue without accumulation in certain brain regions. In contrast, *Wolbachia* of *D*. *paulistorum* (PAU), *D. willistoni* (WIL), and *D. sturtevanti* (STV) were found to be locally restricted (Fig. 1D to F). Although we did not focus on deciphering the identity of infected brain regions in this study, all three species exhibited clear isolation of infection in certain regions of the brain, whereas most of the tissue was free of *Wolbachia*. For measuring *Wolbachia* tropism in respective brains, we determined the restriction indices (RI) as the number of uninfected cells divided by the total number of cells (see Materials and Methods). The indices revealed two significantly distinct groups of either systemic (MEL, SPT, and TRO hosts) or restricted (PAU, WIL, and STV hosts) infections (Fig. 1M), with RI ranging from 0.02 to 0.12 and 0.82 to 0.88, respectively (Poisson regression, *P < *0.001).

10.1128/mbio.03863-21.10 DATA SET S1 Raw data file. Download Data Set S1, XLS file, 0.2 MB. Copyright © 2022 Strunov et al. 2022 Strunov et al. https://creativecommons.org/licenses/by/4.0/ This content is distributed under the terms of the Creative Commons Attribution 4.0 International license.

Next, we examined the distribution of *Wolbachia* in the central nervous system (CNS) of 3rd-instar larvae. The analysis of bacterial infection in larvae of all six species (Fig. 1G to L) using the same FISH approach demonstrated results similar to those obtained for the adult brains. The larval nervous tissue from MEL, SPT, and TRO showed systemic infection (Fig. 1G to I), whereas *Wolbachia* in PAU, WIL, and STV were locally restricted (Fig. 1J to L). Evaluation of the RI for *Wolbachia* infection revealed a limited restriction of bacteria in SIT species in which the index ranged from 0.01 to 0.09. Conversely, the high indices in RIT species ranged from 0.80 to 0.92 (Fig. 1N; Poisson regression, *P < *0.001). Hence, the pattern of bacterial localization is already determined in the larvae and preserved through metamorphosis.

The nervous system of 3rd-instar larvae consists of three different cell types, i.e., neuroblasts (neural stem cells), neurons, and glial cells (22). We therefore asked whether the endosymbiont targets any of these cell types specifically or acts regardless of the lineage in a locally restricted manner. Using a neuroblast-specific antibody against Deadpan, a transcriptional repressor responsible for maintenance of neuroblast’s self-renewing, and also a glia-specific antibody against Repo, a transcriptional factor expressed in glial cells, we analyzed the cell type specificity of *Wolbachia* localization in the CNS of larvae of all six lines (see Fig. S1 in the supplemental material).

10.1128/mbio.03863-21.1 FIG S1 *Wolbachia* infection in neuroblasts of the CNS of 3rd-instar *Drosophila* larvae. Sequential RNA-FISH using *Wolbachia*-specific 16S rRNA probe (red) followed by immunofluorescent staining with anti-Repo (glial cells, green) and anti-Deadpan (neuroblasts, cyan) antibodies of 3rd-instar larval CNS. DNA is stained with DAPI (blue) and actin with phalloidin (green). For each *Drosophila* species, 10 organs were analyzed. Scale bar, 20 μm. Download FIG S1, JPG file, 0.9 MB. Copyright © 2022 Strunov et al. 2022 Strunov et al. https://creativecommons.org/licenses/by/4.0/ This content is distributed under the terms of the Creative Commons Attribution 4.0 International license.

We found infections of glial cells located in the cortex of the CNS in all six analyzed species. MEL, SPT, and TRO showed systemic patterns, whereas bacteria in PAU, WIL, and STV were locally restricted (Fig. S2A). The majority of bacteria, however, were concentrated in neuroblasts and neurons of the larval CNS. Neuroblasts, which we differentiated from other cell types by their bigger size of approximately 10 μm in diameter (see the insets of Fig. 1G to L), showed distinctive *Wolbachia* infection patterns depending on the species analyzed (Fig. S2B). Bacterial densities in a single neuroblast were quantified by dividing the bacterial load within the cell by the area of the cell’s cytoplasm (Fig. S2B). The highest accumulation of bacteria in neural stem cells was observed in MEL and STV, with both densities equating to 0.76. In contrast, TRO and SPT exhibited the lowest densities of 0.13 and 0.30, respectively. Unlike these species, the densities in neuroblasts of PAU and WIL showed an unusually high variance within individual larval CNS, ranging from either 0.2 to 0.79 (mean, 0.51) or 0.1 to 0.79 (mean, 0.57), respectively. High variance in these two restricting hosts suggests that their respective *Wolbachia* strains only target a specific, undetermined subset of neuroblasts. Quantification of RI of bacteria in neuroblasts of all six host species (Fig. 1O) revealed that despite the SIT patterns in MEL and TRO, approximately only half of their neural stem cells were infected with *Wolbachia*, whereas in SPT almost all neuroblasts were *Wolbachia* positive (0.63, 0.51, and 0.02; Poisson regression, *P < *0.001). On the other hand, all hosts with RIT patterns (PAU, WIL, and STV) showed significantly higher RIs than systemic ones (0.95, 0.93, and 0.92; Poisson regression, *P < *0.001).

10.1128/mbio.03863-21.2 FIG S2 *Wolbachia* infection of glial cells and density and aggregation of *Wolbachia* in the CNS of 3rd-instar *Drosophila* larvae. (A) Sequential FISH using *Wolbachia*-specific 16S rRNA probe (red) followed by immunofluorescent staining with anti-Repo (glial cells, green) and anti-Deadpan (neuroblasts, cyan). DNA is stained with DAPI (blue) and actin with phalloidin (green). Asterisks indicate a glial cell infected with *Wolbachia*. For each *Drosophila* species 10 organs were analyzed. (B) Density within 10 neuroblasts of 3 individual brains quantified with Fiji as a bacterial load area divided by an area of cell cytoplasm. (B) Aggregation of infection in the larval CNS of six *Drosophila* species analyzed from bacterial clusters in 5 individual brains (61 to 65 clusters for SIT and 26 to 32 clusters for RIT) by quantifying the number of neighboring infected neurons in groups. Asterisks denote statistical significance (*, *P < *0.05; ***, *P < *0.001; one-way ANOVA with Tukey HSD test). (C) Statistical significance is shown with letters (*P < *0.05, one-way ANOVA with Tukey HSD test). Red bars show standard deviations, red dots designate the mean value. For more details, see the supplemental material. Scale bar, 10 μm. Download FIG S2, JPG file, 1.7 MB. Copyright © 2022 Strunov et al. 2022 Strunov et al. https://creativecommons.org/licenses/by/4.0/ This content is distributed under the terms of the Creative Commons Attribution 4.0 International license.

By using a specific antibody against Asense, a transcriptional factor expressed in type I but not type II neuroblasts, we further specified the cell type of infection (Fig. S3). Type II neuroblasts divide symmetrically, producing intermediate neural progenitors, which then divide asymmetrically to self-renew and generate a ganglion mother cell, whereas type I neuroblasts divide asymmetrically and only once (22). As a result, type II neuroblasts generate a greater number of cells in the adult brain than type I. We hypothesized that infecting type II neuronal stem cells is an opportunity for *Wolbachia* to achieve a broader spread. In all three species with SIT pattern, *Wolbachia* were present in both neuroblast types (Fig. S3, first 3 rows). For hosts with RIT patterns, however, only type I neuroblasts were found infected with the endosymbiont (Fig. S3, last 3 rows).

10.1128/mbio.03863-21.3 FIG S3 *Wolbachia* infection in type I and II neuroblasts in the CNS of 3rd-instar *Drosophila* larvae. Sequential FISH using *Wolbachia*-specific 16S rRNA probe (green dots) and immunofluorescent staining with anti-Asense antibody (red), which is diagnostic for type I neuroblasts of 3rd-instar larval CNS. DNA is stained with DAPI (blue), actin with phalloidin (green). Asterisks depict type II neuroblasts, which are Asense negative, infected with *Wolbachia* (green dots). In total 10 brains were analyzed for each species. Scale bar, 20 μm (MEL, SPT, and TRO) and 10 μm (PAU, WIL, and STV). Download FIG S3, JPG file, 1.0 MB. Copyright © 2022 Strunov et al. 2022 Strunov et al. https://creativecommons.org/licenses/by/4.0/ This content is distributed under the terms of the Creative Commons Attribution 4.0 International license.

Furthermore, to analyze the aggregation of *Wolbachia* infection in the CNS, i.e., the formation of clusters of neighboring neurons bearing infections, we quantified the average number of infected neurons in groups (Fig. S2C). Quantifications demonstrated the formation of big clusters of infected neurons in SPT, MEL, and STV (21.1, 18.5, and 15.9 neurons on average per cluster, respectively) and smaller clusters in WIL, TRO, and PAU (13.5, 9.5, and 7.2 neurons on average per cluster, respectively) without statistically significant differences between systemic and restricting hosts (*P > *0.05).

In summary, we observe two distinct patterns of *Wolbachia* tropism in *Drosophila* nervous tissues, the systemic in MEL, SPT, and TRO, with an overall distribution of infection, and the restricted in PAU, WIL, and STV, with isolation of infection to certain areas of the tissue. These data strongly imply that the pattern of infection is already determined in 3rd-instar larvae and preserved through metamorphosis with no tropism to a specific type of nerve cell but dominating at higher densities in neuroblasts, the neural stem cells. To screen more saltans group representatives, *Wolbachia* FISH in neuronal tissues of *D. lehrmanae* (sturtevanti subgroup) and *D. prosaltans* (saltans subgroup) exhibited, similar to STV and SPT hosts, either restricted (Fig. S4A and B) or systemic patterns (Fig. S4C and D), respectively. Interestingly, bacterial densities within neural stem cells as well as their ability to aggregate vary among different *Drosophila* hosts irrespective of their diagnostic SIT and RIT patterns.

10.1128/mbio.03863-21.4 FIG S4 *Wolbachia* infection in nervous tissues of *Drosophila lehrmanae* (A and B) from sturtevanti subgroup and *Drosophila prosaltans* (C and D) from saltans subgroup and densities of *Wolbachia* in the nurse cells of stage 3 to 5 ovaries of neotropical *Drosophila* species. (A to D) Fluorescent *in situ* hybridization on 3rd-instar larval CNS (A and C) and adult brains (B and D) using 16S rRNA *Wolbachia*-specific probe (red). DNA is stained with DAPI (blue). Note restriction of *Wolbachia* in *D. lehrmanae* and systemic infection in *D. prosaltans*. (E) The bacterial density was analyzed in all six *Drosophila* species with Fiji as the bacterial infection area in an egg chamber divided by an area of the chamber. Asterisks denote statistical significance (*, *P < *0.05; **, *P < *0.01; ***, *P < *0.001). Red bars show standard deviations, red dots designate the mean value. In total, 10 egg chambers were analyzed for every species (see Data Set S1). Scale bar, 50 μm. Download FIG S4, JPG file, 1.2 MB. Copyright © 2022 Strunov et al. 2022 Strunov et al. https://creativecommons.org/licenses/by/4.0/ This content is distributed under the terms of the Creative Commons Attribution 4.0 International license.

### Tropism of *Wolbachia* in *Drosophila* ovaries.

**FIG 2:**
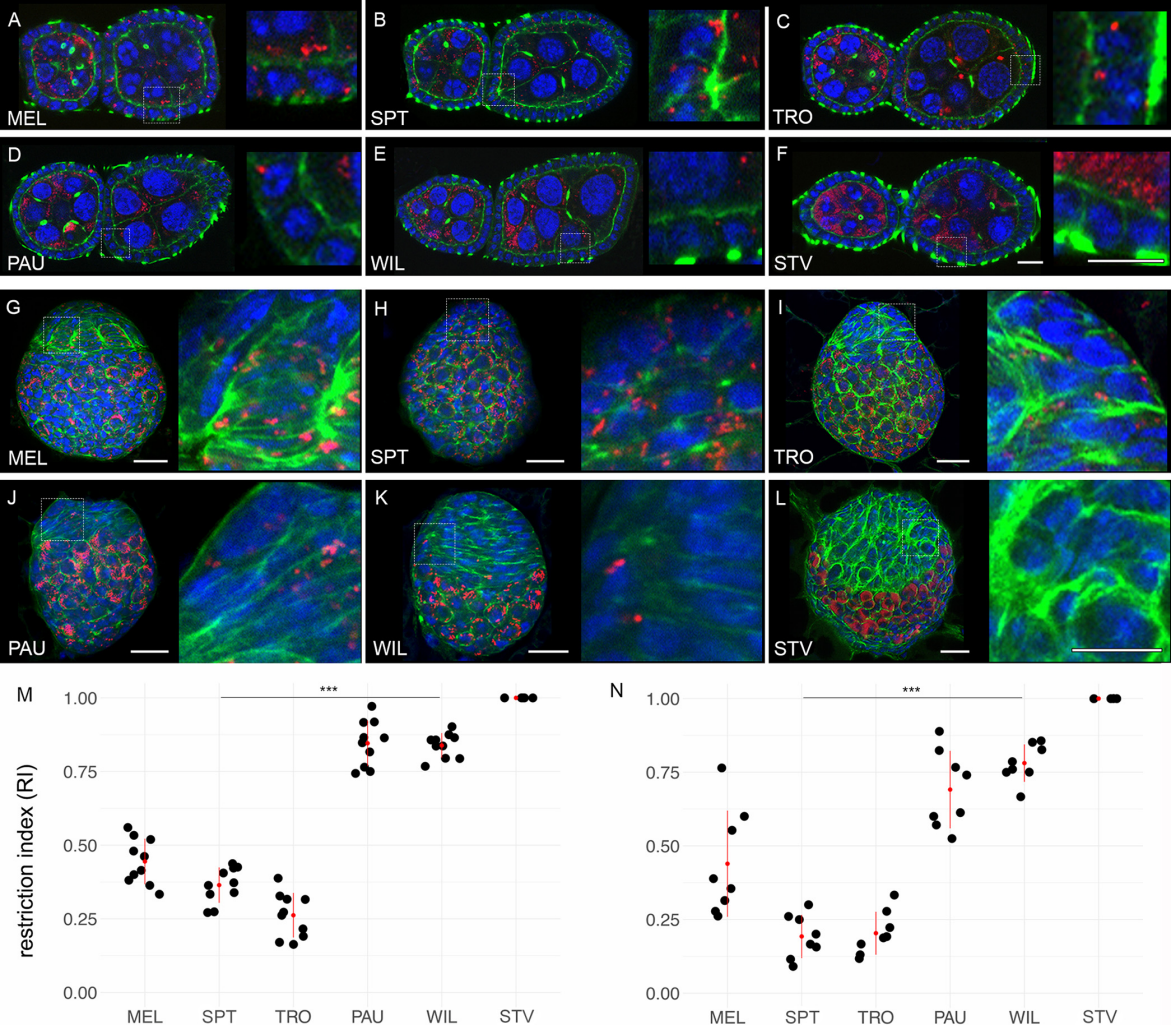
Restriction of *Wolbachia* infection in the soma and the germ line of adult and larval ovaries of neotropical *Drosophila*. Fluorescent *in situ* hybridization of different *Drosophila* adult ovaries (A to F) and 3rd-instar larval ovaries (G to L) using 16S rRNA *Wolbachia*-specific probe (red). RIs of *Wolbachia* infection in follicle cells of adult (M) and larval (N) ovaries for all six species. DNA is stained with DAPI (blue), actin with phalloidin (green). Asterisks denote statistical significance (***, *P < *0.001; Poisson regression). Red bars show standard deviations, red dots designate the mean value. In total, 8 to 10 organs were analyzed for every species (see Data Set S1). Scale bar, 20 μm.

For transovarial transmission, *Wolbachia* endosymbionts need to colonize the female germ line. *Drosophila* ovaries consist of reproductive and somatic tissues. The nurse cells and the oocytes, originating from the germ line stem cells, form the reproductive part. Conversely, the follicle cells, which ensheath the former, are derived from the somatic stem cell niche and represent the somatic part (23). Our systematic analysis of bacterial infections using FISH in the adult ovaries at stage 3 to 5 of the six species revealed that the majority of bacteria are associated with the reproductive part. However, they are also found in the soma but generally at lower levels (Fig. 2A to F).

Infection density, which was quantified as a ratio of bacterial area to the area of interest in the tissue (see Materials and Methods) in nurse cells and the oocyte was significantly higher in PAU, WIL, and STV than in MEL, SPT, and TRO (Fig. S4E; Poisson regression, *P < *0.001). We also observed *Wolbachia* infection in the follicle cells. Respective RIs in follicle cells, quantified as a ratio of uninfected follicle cells to the total number of follicle cells, varied among the species with relatively low average values in the systemic hosts TRO, SPT, and MEL (Fig. 2M) (0.26, 0.36 and 0.44, respectively), but significantly higher in WIL, PAU and STV restrictors (0.84, 0.85 and 1, respectively; Poisson regression, *P < *0.001).

The analysis of bacterial infection using FISH in 3rd-instar larval ovaries revealed similar results, as observed in the adult ovaries (Fig. 2G to L). The larval ovary can also be divided into somatic and reproductive parts either morphologically or by specific staining. Similar to adult ovaries, native *Wolbachia* are dominating in the reproductive part (germ cells) of all six species analyzed. In the somatic part, however, low restriction of infection was observed only in systemic SPT, TRO, and MEL hosts (Fig. 2N) (0.19, 0.20, and 0.44, respectively). In contrast, WIL and PAU exert significantly higher restriction (0.78 and 0.70, respectively; Poisson regression, *P < *0.001), whereas in STV the infection was not detectable at all in the somatic part of the anlage. The preservation of infection patterns in the somatic part of the adult ovary compared to the larval gonad is reminiscent of the pattern described for the larval CNS and adult brain, where the bacterial distribution was also preserved after metamorphosis.

### *Wolbachia* infection of *Drosophila* hemocytes.

Both tissues, brain and ovaries, of RIT species showed confined infection patterns that were already established during larval development and preserved through metamorphosis. To account for the possibility of active migration and dispersion of *Wolbachia* from bacterial isolates all over the body via the hemolymph stream (24), we analyzed the infection status of hemocytes extracted from whole adult bodies of the six species by using *Wolbachia*-specific FISH (Fig. S5A). While all three SIT species showed high rates of bacterial infection, ranging from 65.4% to 90.1%, PAU and WIL hosts had significantly lower rates of 24.6% and 32.3%, respectively (Fig. S5B). On the contrary, 57.1% of hemocytes were infected in STV, which ranges between SIT and RIT levels and, hence, does not follow this global trend (Fig. S5B). Importantly, *Wolbachia* of *D. sturtevanti* are quite distantly related to the *w*Au-like infections of the other neotropical willistonii and saltans group hosts (18, 25). This indicates a more recent infection of *D. sturtevanti* flies from an outside source, whereas *w*Au-like variants of neotropical hosts are usually conspecific (18). Together, differences in evolutionary histories might account for the intermediate phenotype observed in *w*Stv-infected hemocytes, whereas the partial restriction in PAU and WIL hemocytes can be explained by the possibility that RIT are limited to some defined immune cell subtypes only, similar to type I and type II neuroblasts (Fig. S3). The functional bases of such cell type specificities are unknown and represent a very intriguing question, which we currently aim to answer in more detail in our laboratory.

10.1128/mbio.03863-21.5 FIG S5 *Wolbachia* infection of *Drosophila* hemocytes from systemic and restricted species. (A) Fluorescent *in situ* hybridization on isolated hemocytes of adult using 16S rRNA *Wolbachia*-specific probe (red). Actin is stained with phalloidin (green). DNA is stained with DAPI (blue). (B) Ratio of infected hemocytes in 6 *Drosophila* species analyzed; 30 to 40 hemocytes were counted per species. Download FIG S5, JPG file, 0.4 MB. Copyright © 2022 Strunov et al. 2022 Strunov et al. https://creativecommons.org/licenses/by/4.0/ This content is distributed under the terms of the Creative Commons Attribution 4.0 International license.

### *Wolbachia* densities drop dramatically during early embryonic gastrulation in *Drosophila* species with restricting pattern of infection.

**FIG 3:**
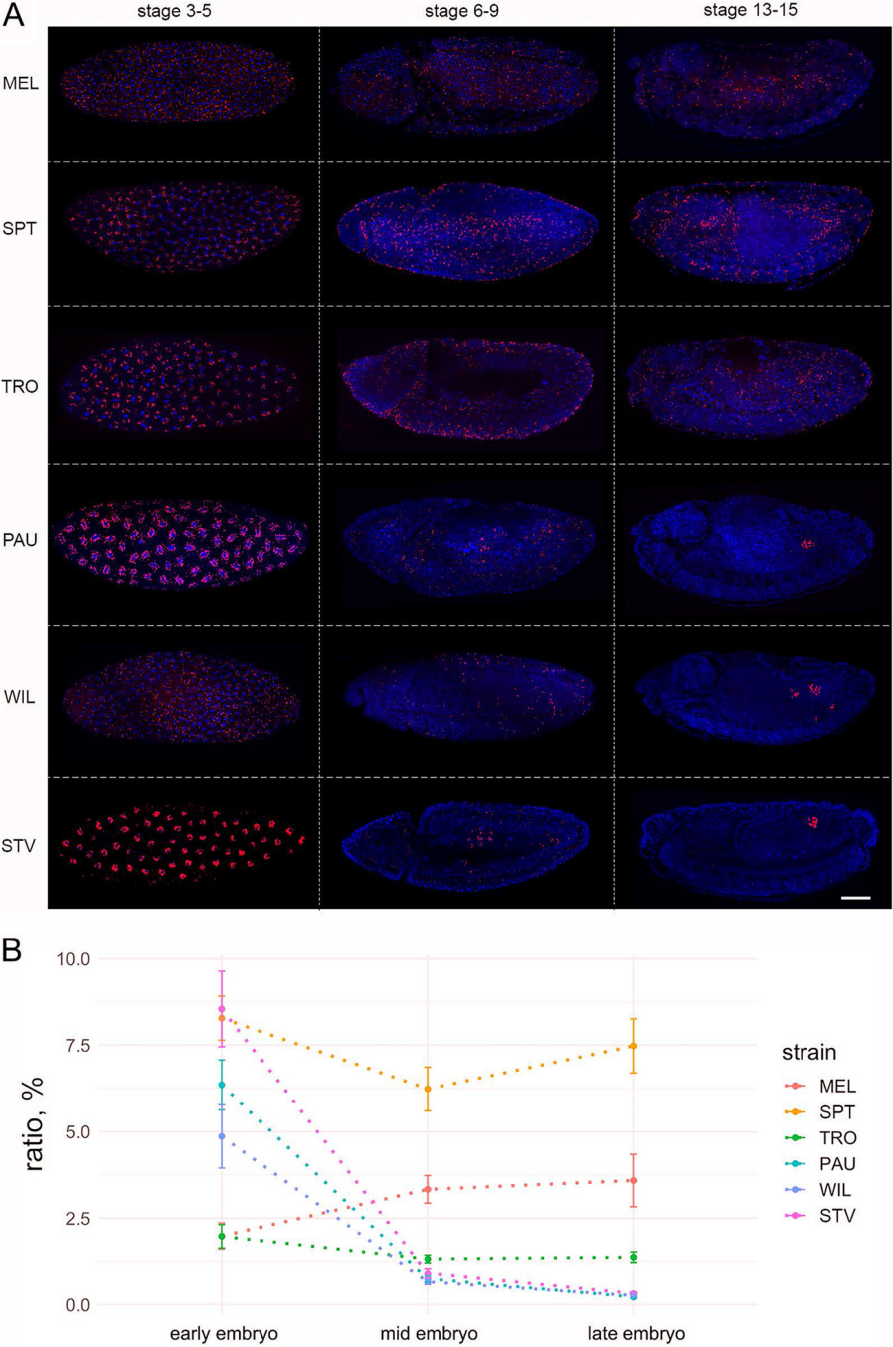
Dramatic reduction of *Wolbachia* density during mid-embryogenesis in neotropical *Drosophila* species. (A) Fluorescent *in situ* hybridization of *Drosophila* embryos at stages 3 to 5, 6 to 9, and 13 to 15 of embryogenesis, using 16S rRNA *Wolbachia*-specific probe (red). DNA is stained with DAPI (blue). (B) Quantification of *Wolbachia* density at early, mid-, and late embryogenesis, using Fiji, as bacterial density in a whole embryo divided by the area of an embryo. Bars show standard errors of the means. For each species and stage, 5 embryos were analyzed for density measurements (see Data Set S1). Scale bar, 50 μm.

Data obtained from the adult and 3rd-instar larval soma and germ line demonstrate that cell type-specific tropisms of *Wolbachia* are determined already in larvae and are preserved during the metamorphosis of the host. To investigate how infection patterns form initially, we analyzed *Wolbachia* distribution through different *Drosophila* embryogenesis stages. Analysis of *Wolbachia* localization in early embryos (stages 3 to 5) revealed SIT patterns with no differences in infection distribution in any of the six tested hosts (Fig. 3, left row). Bacteria were evenly dispersed all over the embryo and closely associated with chromatin during mitosis. Interestingly, in mid-embryogenesis (stages 6 to 9), *Wolbachia* densities decreased in PAU, WIL, and STV but not in MEL, SPT, and TRO embryos (Fig. 3A, middle row). Although bacteria were still evenly distributed across all embryonic areas in all six species at early gastrulation, many cells of PAU, WIL, and STV embryos were already cleared of infection. Finally, at late embryogenesis (stages 13 to 15), we observed drastic differences in *Wolbachia* distribution between species with SIT and RIT patterns of bacterial infection (Fig. 3, right row). While in systemic MEL, SPT, and TRO hosts bacteria were equally dispersed in most embryonic tissues, *Wolbachia* in PAU, WIL, and STV species were now locally restricted to the primordial germ cells (PGCs), the future gonads, and some additional isolated somatic cell clusters in the embryo.

Quantification of global *Wolbachia* densities in embryos at these three defined developmental stages using Fiji confirmed this dramatic reduction of infection starting at mid-embryogenesis in PAU, WIL, and STV (*P < *0.001, one-way analysis of variance [ANOVA] with Tukey honestly significant difference [HSD] test), whereas densities of bacteria in MEL, TRO, and SPT hosts remained unchanged across all stages (Fig. 3B).

**FIG 4:**
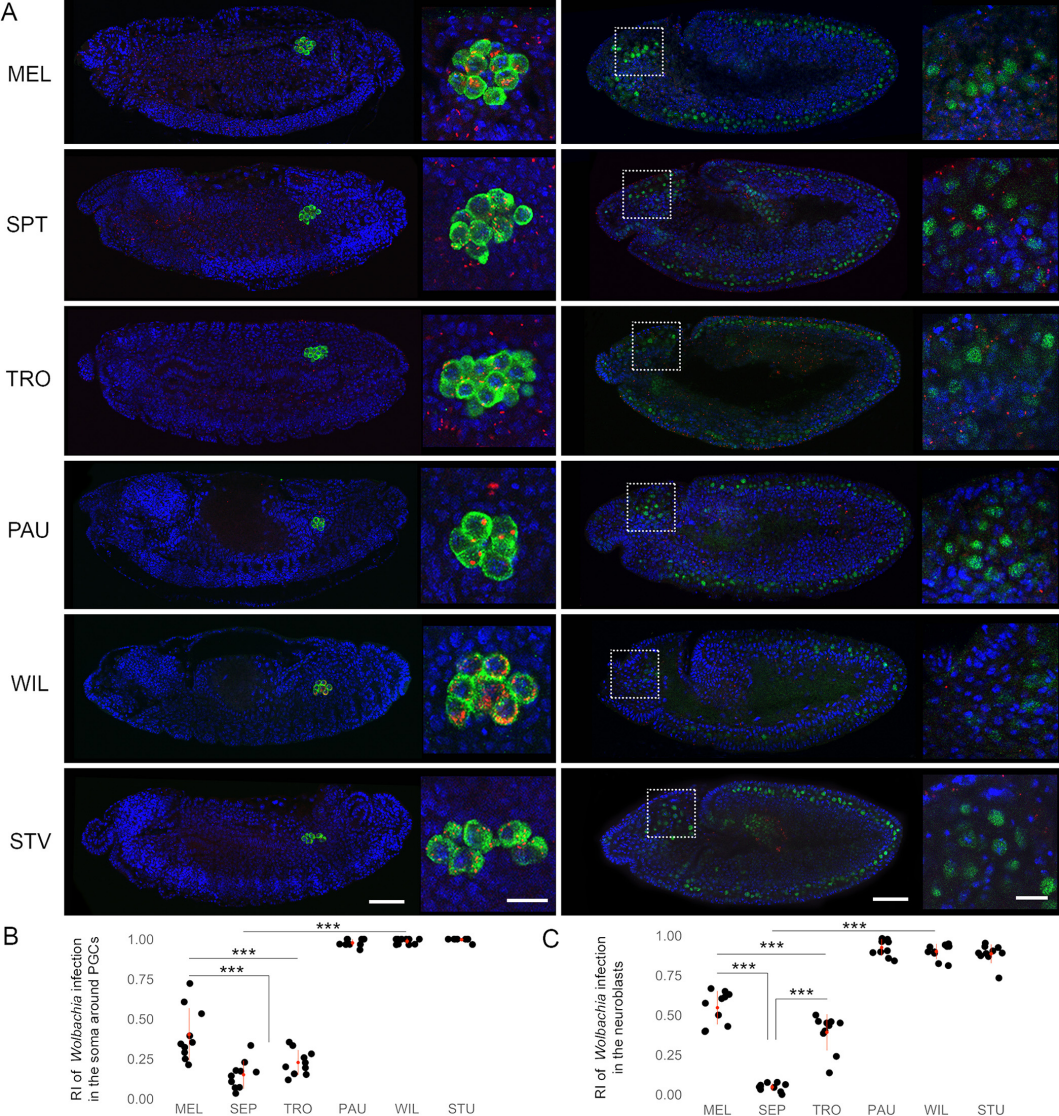
*Wolbachia* tropism to primordial germ cells and neuroblasts of neotropical *Drosophila* embryos. (A) Sequential FISH using *Wolbachia*-specific 16S rRNA probe (red) and immunofluorescent staining of PGCs with anti-Vasa (left column, green) and neuroblasts with anti-Deadpan (right column, green) antibodies on *Drosophila* embryos. DNA is stained with DAPI (blue). (B and C) Determined RIs in the soma of neighboring PGCs (B) and in neuroblasts (C). In total, 10 embryos were analyzed for every cell type (see Data Set S1)). Asterisks denote statistical significance (***, *P < *0.001; one-way ANOVA with Tukey HSD test). Red bars show standard deviations, red dots designate the mean value. Scale bar, 50 μm for embryos, 10 μm for insets.

To further test our hypothesis that *Wolbachia* are selectively maintained mainly in PGCs of late WIL, PAU, and STV embryos, we performed sequential FISH and immunofluorescence analysis using an antibody against Vasa, a protein essential for the pole plasm assembly in the egg, a commonly used germ line precursor marker (26). As expected from a maternally transmitted endosymbiont, all six tested host species harbored the bacterial infection within their PGCs (Fig. 4A, left column). However, only PAU, WIL, and STV hosts showed strict isolation of infection within the PGCs with infrequent bacterial localization in surrounding soma, whereas in MEL, SPT, and TRO *Wolbachia* remained systemic (*P < *0.001; one-way ANOVA with Tukey HSD test) (Fig. 4B).

Additionally, using a similar approach but with the neuroblast-specific Deadpan antibody, we analyzed bacterial tropism in embryonic neuroblasts after their delamination from the neuroectoderm at stages 9 to 10 (Fig. 4A, right column). Similar profound elimination of bacteria from the soma (neuroblasts in this case) was observed in PAU, WIL, and STV species, in contrast to an ongoing systemic infection in MEL, SPT, and TRO (*P < *0.001; one-way ANOVA with Tukey HSD test). Already after delamination of the neuroblasts in procephalic neurogenic region, which gives rise to the brain of an embryo, we detected only a very few nuclei associated with *Wolbachia* signals in species restricting the infection, whereas at least half of the neuroblasts of SIT hosts contained the bacteria (Fig. 4A, right column insets, and C).

In summary, by systematically tracing the temporal and spatial dynamics of *Wolbachia* tropism *in situ*, we found that bacterial densities started to drop already before gastrulation (stages 6 to 9) exclusively in three RIT species. The majority of *Wolbachia* accumulated mainly in PGCs but also in a few other cells of the embryo (neuroblasts and other undefined cell types). Hence, restricted *Wolbachia* tropism found in the germ line and the soma of PAU, WIL, and STV hosts is already determined before the onset of gastrulation.

### Autophagy eliminates *Wolbachia* in restricting species during early gastrulation.

**FIG 5:**
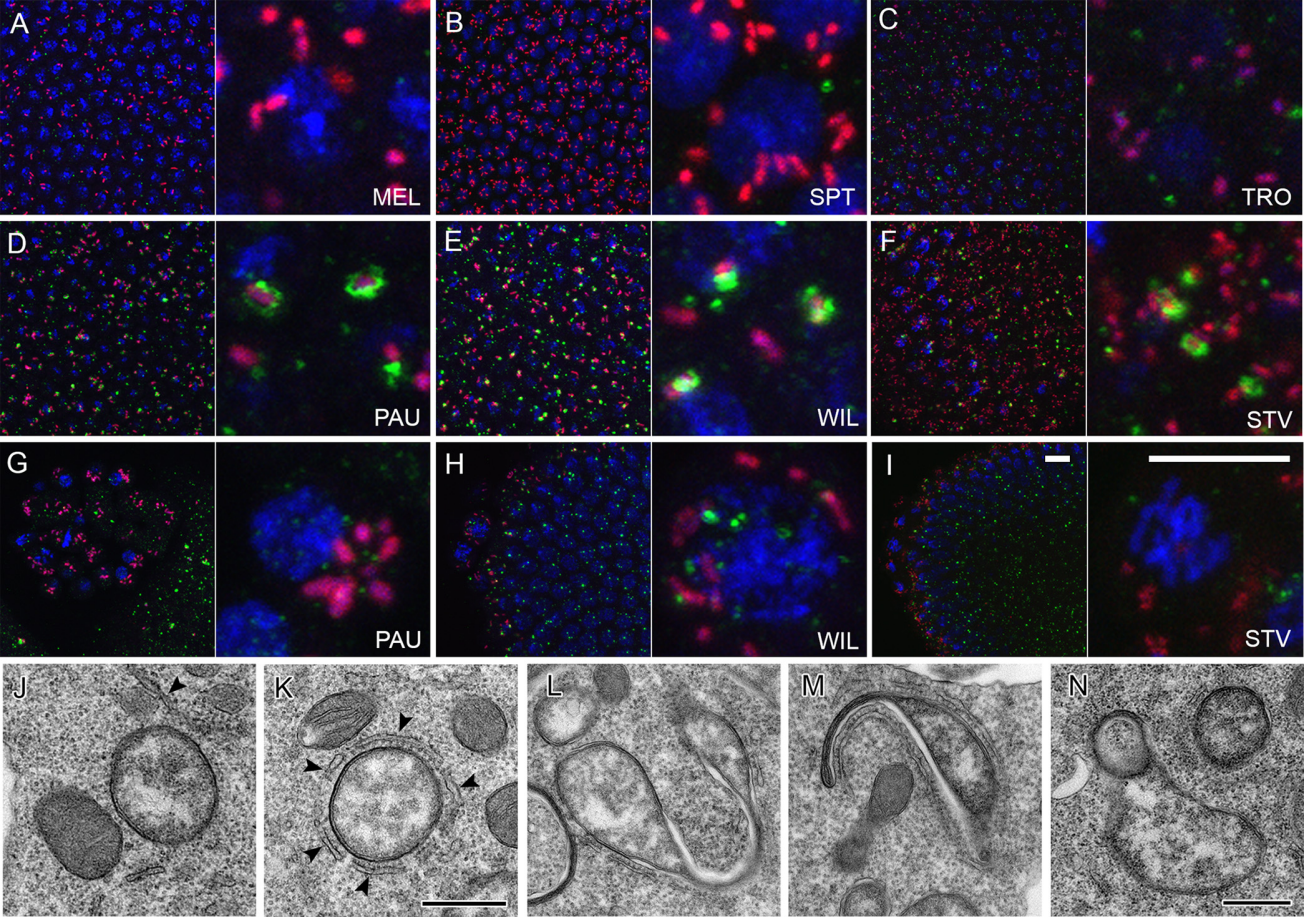
Elimination of *Wolbachia* via autophagy in neotropical *Drosophila* embryos. Sequential FISH using *Wolbachia*-specific 16S rRNA probe (red) and immunofluorescent staining with anti-GABARAP (green) antibody of embryos at stage 5 (A to I). Note the absence of autophagy in SIT species (A to C) and formation of autophagosomes (green rings) around *Wolbachia* in RIT species (D to F). Also note the absence of autophagy in PGCs of RIT species (G to I). (J and K) Transmission electron microscopy on systemic MEL (J) and restrictive PAU (K) embryos at the cellularization and early gastrulation (stage 5 and 6). Contrary to MEL (J), tight physical associations between *w*Pau *Wolbachia* and the endoplasmic reticulum of restrictive PAU hosts (arrowheads) are prominent (K). (L to N) Abnormal *w*Pau *Wolbachia* morphotypes with signs of stretching (L), membrane extrusions (M), and vesicle formation (N). DNA is stained with DAPI (blue). Scale bar, 10 μm for all fluorescent images, 0.5 μm for TEM.

Since we detected a dramatic decrease in bacterial titer already during embryogenesis (Fig. 3B), we hypothesized that active host-directed elimination of the endosymbiont is a more plausible mechanism of infection restriction than dilution and/or selective replication. Autophagy was considered a potential mechanism for bacterial clearance because it has previously been demonstrated as a key cellular strategy for controlling *Wolbachia* density and tropism in Brugia malayi nematodes and D. melanogaster flies *in vivo* as well as *in vitro* in cell lines of D. melanogaster and Aedes albopictus (27). Moreover, it was recently shown that the density of *Wolbachia* in D. melanogaster is mediated by host autophagy in a cell type-dependent manner (28). To test our hypothesis, we conducted sequential FISH and immunofluorescent analysis using an anti-GABARAP antibody, which is diagnostic for maturing autophagosomes in a cell. Since the drastic loss of somatic *Wolbachia* was clearly evident at mid-embryogenesis of restricted hosts (stages 6 to 9) (Fig. 3A, middle row), we focused our analysis on early to late blastodermal embryos to study the temporal and spatial dynamics of the elimination process *in situ*. No signs of bacterial autophagy were found in the soma or in PGCs of systemic MEL, SPT, and TRO hosts (Fig. 5A to C and Fig. S6A), whereas in the soma of restricted PAU, WIL, and STV embryos we observed the formation of GABARAP-positive rings around bacterial cells (Fig. 5D to F). The earliest cases of *Wolbachia* engulfment were found in blastodermal embryos (stage 5), with the highest peak in early gastrulation (stage 6) and only rarely at later stages (stages 7 to 8). Importantly, PGCs, which could be recognized as an isolated cell cluster at posterior part of the embryo in late blastodermal or early gastrulating embryo, were devoid of any signs of bacterial autophagy in all three RIT species (Fig. 5G to I). This was in full agreement with our observations from later embryos: here, *Wolbachia* are preserved and maintained in the gonad precursor cells (Fig. 4A, left column).

10.1128/mbio.03863-21.6 FIG S6 *Wolbachia* colocalization with anti-GABARAP and anti-FK2 antibodies. (A) Absence of colocalization of *Wolbachia* with anti-GABARAP and anti-FK2 in primordial germ cells of *Drosophila* with SIT pattern of infection at stage 5 of embryogenesis. Sequential FISH using *Wolbachia*-specific 16S rRNA probe (red) and immunofluorescent staining with the two autophagy-specific antibodies, i.e., anti-GABARAP (green, upper) and anti-FK2 (green, lower) on PGCs of embryos from the six species at the cellularization stage. DNA is stained with DAPI in blue. For each *Drosophila* species five embryos were analyzed. (B and C) *Wolbachia* colocalization with anti-GABARAP (B) and anti-FK2 (C) antibodies in 6 *Drosophila* species analyzed. Colocalization was assessed using the JACoP Fiji plugin. Each dot represents percentage of colocalization in a single embryo in the soma at stages 3 to 4 and stages 5 to 6 or PGCs of both (stages were fused due to the absence of differences). In panel B asterisks denote statistical significance only for soma of early-mid embryo at stages 5 to 6 (***, *P < *0.001; one-way ANOVA with Tukey HSD test). In panel C letters indicate statistical significance only for soma of early-mid embryo at stages 5 to 6 (*P < *0.001, one-way ANOVA with Tukey HSD test). X demonstrates the mean value. For every species and every stage, 4 to 11 embryos were analyzed (see the supplemental material). (D and E) *Wolbachia* colocalization with anti-GABARAP (D) and anti-FK2 (E) antibodies in transinfected line. Colocalization was assessed the same way. Asterisks denote statistical significance only for soma of early-mid embryo at stage 6 (***, *P < *0.001; one-way ANOVA with Tukey HSD test). For every species and every stage, 4 to 11 embryos were analyzed (see Data Set S1). Scale bar, 20 μm. Download FIG S6, JPG file, 0.7 MB. Copyright © 2022 Strunov et al. 2022 Strunov et al. https://creativecommons.org/licenses/by/4.0/ This content is distributed under the terms of the Creative Commons Attribution 4.0 International license.

To further support our observation, we quantified the colocalization of GABARAP and *Wolbachia* cells using a JACoP plugin (29) for the imaging software Fiji (30). We found a pronounced overlap of autophagosomes and *Wolbachia* in the soma of the blastodermal and early gastrulating embryos (stages 5 to 6) of PAU, WIL, and STV species, with 22.3% ± 2.2%, 25.8% ± 3.4%, and 15.5% ± 4.1%, respectively. In contrast, in the soma of earlier embryos (stages 3 to 4) and PGCs at both developmental stages of all six species, we detected significantly less colocalization (between 0 and 2%) of *Wolbachia* with the antibody (Poisson regression, *P < *0.001), confirming that there is no clearance of bacterial infection at this stage (Fig. S6B).

To further decipher the mechanistic basis of these intimate bacterial interactions with autophagosomes, we conducted an ultrastructural analysis of MEL and PAU embryos at cellularization and early gastrulation stages. Transmission electron microscopy (TEM) of PAU embryos at these stages revealed intimate interaction of *Wolbachia* with the endoplasmic reticulum (ER) of the host cell, contrary to MEL species, where no similar types of tight associations were detected (Fig. 5J and K). In most of the cases we observed rough ER membranes encircling the bacterial cells by close apposition but without direct contact (Fig. 5K). Later in early gastrulating PAU embryos, abnormal *Wolbachia* bacteria are dominant, exhibiting various signs of stretching, membrane extrusions, and vesicle formation (Fig. 5L to N and Fig. S7A to C) that indicate symbiont degradation. No such structures were observed in MEL embryos at this stage. Surprisingly, we did not observe any autophagosome-like structures or traces of lysed bacteria at cellularization and early gastrulation, which is in contrast to clear colocalization of anti-GABARAP antibody and *Wolbachia* obtained with sequential FISH and immunofluorescent staining (Fig. 5D and Fig. S6B). The most plausible explanation for this observation is that autophagy of bacteria occurs in a noncanonical way. The abnormal *Wolbachia* forms we detected in early gastrulating embryos of restricting species support this hypothesis.

10.1128/mbio.03863-21.7 FIG S7 *Wolbachia* interactions with the host cell. Transmission electron microscopy images of abnormal *Wolbachia* in early gastrulating PAU embryos in the soma (A to C) demonstrating abnormalities in morphology like vesicle formation (A), stretching (B), and membrane extrusions (C). (D to L) Sequential FISH using *Wolbachia*-specific 16S rRNA probe (red) and immunofluorescent staining with anti-FK2 (green) antibody. Note the absence of ubiquitination in SIT species (D to F) and colocalization of anti-FK2 with *Wolbachia* in RIT species (G to I). Also note the absence of colocalization of anti-FK2 with bacteria in PGCs of restricting species (J to L). Scale bar: 0.1 μm (A to C), 10 μm (D to L). Download FIG S7, JPG file, 1.1 MB. Copyright © 2022 Strunov et al. 2022 Strunov et al. https://creativecommons.org/licenses/by/4.0/ This content is distributed under the terms of the Creative Commons Attribution 4.0 International license.

Besides anti-GABARAP, we also used an anti-FK2 antibody that recognizes mono- and polyubiquitinated conjugates to decipher whether bacteria are tagged for subsequent degradation. Consistent with our previous observations with anti-GABARAP staining, we did not detect any signs of ubiquitination of *Wolbachia* in MEL, SPT, and TRO embryos at blastodermal and gastrulating stages (Fig. S7D to F), including the PGCs (Fig. S6A). Furthermore, we did not detect frequent colocalization of anti-FK2 antibody and *Wolbachia* in PAU and STV embryos at both embryonic stages (Fig. S7G to I and Fig. S6C). Surprisingly, only WIL embryos exhibited pronounced ubiquitination signals associated with *Wolbachia* already at the blastodermal stage of embryogenesis (Fig. S7H and Fig. S6C). The signal from the antibody staining was confined to half of the bacterial surface, in contrast to the ring-like structures observed with anti-GABARAP (Fig. 5E). Similar to anti-GABARAP staining, no colocalization of *Wolbachia* with anti-FK2 antibody was found in PGC of all six species (Fig. S6A and Fig. S7J to L).

To test for the active elimination of *Wolbachia* in larval and adult tissues after embryogenesis, we analyzed bacterial autophagy in the central nervous tissue of 3rd-instar larvae and brains and ovaries of 1-week-old adult flies of the six *Drosophila* species. All three tissues (6 individuals analyzed each) exhibited only very rare cases of autophagosome formation around *Wolbachia* cells (two bacterial cells per confocal section of the whole organ) (Fig. S8). These data demonstrate that there is no substantial regulation of bacterial infection via autophagy in larvae and adult flies and that *Wolbachia*-specific autophagy for active clearing of infection is restricted to early embryogenesis. Apart from autophagy, there might be other mechanisms constraining the infection, which is an aim of our future project in the lab.

10.1128/mbio.03863-21.8 FIG S8 Summary of sequential FISH using *Wolbachia*-specific 16S rRNA probe (red) and immunofluorescent staining with anti-GABARAP (green) antibody of adult brains, adult ovaries and larval CNS. Actin is stained with phalloidin (green). Nuclei are stained with DAPI (blue). Note rare cases of *Wolbachia* encirclement with anti-GABARAP antibody. Six organs were analyzed per tissue per species. Download FIG S8, JPG file, 0.8 MB. Copyright © 2022 Strunov et al. 2022 Strunov et al. https://creativecommons.org/licenses/by/4.0/ This content is distributed under the terms of the Creative Commons Attribution 4.0 International license.

To sum up, analysis of blastodermal and early gastrulating embryos revealed that massive *Wolbachia* reduction in the tissues of restricting hosts is connected to autophagy mediated by the intimate interactions of bacteria with ER membranes of the host cell. This occurs only in early embryogenesis and was not observed at later stages of host development. Interestingly, while *w*Wil bacteria exhibited the interaction with ubiquitin, the two other native endosymbionts of PAU and STV did not show any signs of ubiquitination. The mechanistic basis of the observed differences awaits further studies in our laboratory.

### Host background plays a major role in regulating the pattern of *Wolbachia* tropism in the soma.

**FIG 6:**
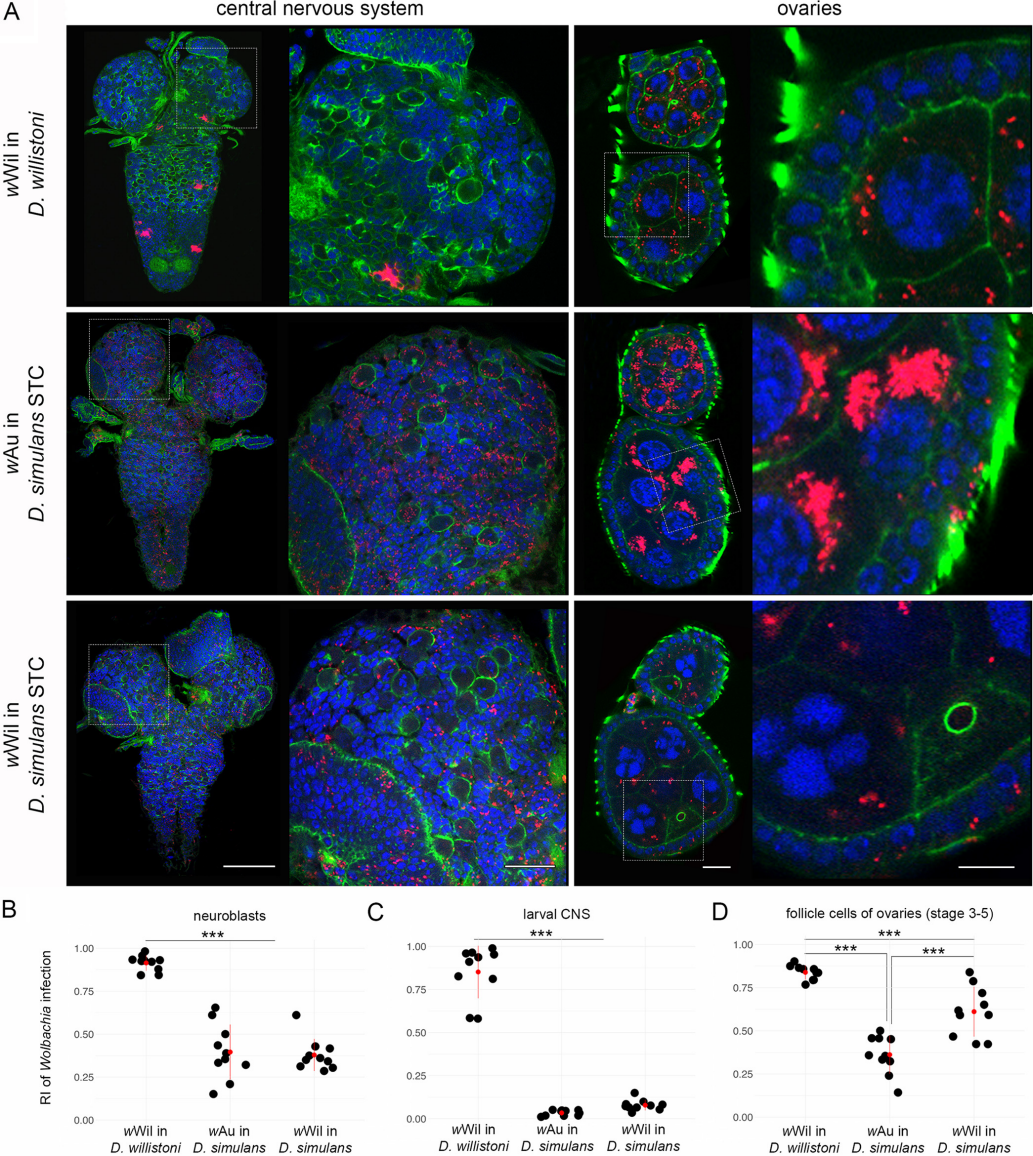
Tropism of the restrictive *w*Wil strain from *D. willistoni* in systemic *D. simulans* host. (A) Fluorescent *in situ* hybridization of different *Drosophila* 3rd-instar larval CNS (left column) and adult ovaries at stages 3 to 5 (right column) of *D. willsitoni*, *D. simulans*, and *D. simulans* transinfected with *w*Wil strain using 16S rRNA *Wolbachia*-specific probe (red). (B) The RI of bacteria in neuroblasts. (C and D) RIs of *Wolbachia* infection in the larval CNS and follicle cells of adult ovaries. DNA is stained with DAPI (blue); actin is stained with phalloidin (green). For each *Drosophila* species, 10 organs from each developmental stage were analyzed (see Data Set S1). Asterisks denote statistical significance (***, *P < *0.001; one-way ANOVA with Tukey HSD test). Red bars show standard deviations, red dots designate the mean value. Scale bar, 20 μm.

To test the influence of each partner in this intimate symbiotic association, we conducted experiments with transinfected flies carrying different *Wolbachia* strains in the same host background. Drosophila simulans flies that are naturally infected with *Wolbachia* strains like *w*Au or *w*Ri, demonstrating the SIT, were first cleared from the infection using antibiotics (now named *D. simulans* STC) and subsequently transinfected with *w*Wil strain from *D. willistoni* via embryonic microinjections. Thus, a *Wolbachia* strain accommodated to the restricting host background was introduced into the SIT environment. In our experiment, the successfully transinfected line *w*Wil/STC was kept in the lab for more than 10 years before starting further analyses on symbiont tropism in the *de novo* host background. Comparative FISH analysis of 3rd-instar larval CNS and adult ovaries (stages 3 to 5) with *Wolbachia*-specific probes showed that the *de novo w*Wil infection in *D. simulans* is not restricted as it is in *D. willistoni* but is systemic, similar to the globally dispersed patterns when infected with their natural strains of *Wolbachia* (Fig. 6A).

**FIG 7:**
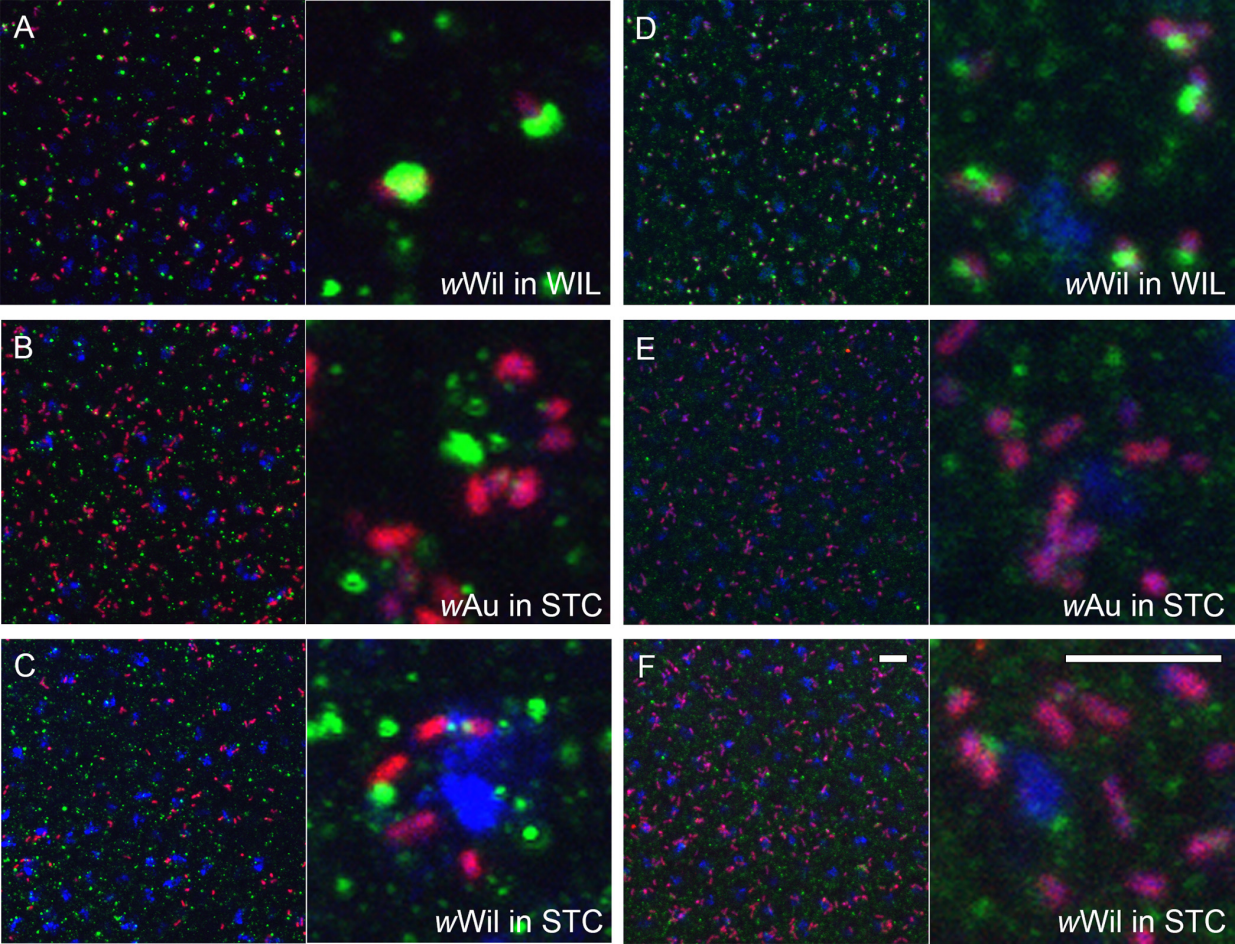
*Wolbachia* interactions with the host cell. Sequential FISH using *Wolbachia*-specific 16S rRNA probe (red) and immunofluorescent staining with anti-GABARAP (A to C) and anti-FK2 (D to F) antibodies on stage 6 embryos from *D. willsitoni* (*w*Wil in WIL), natively *w*Au-infected *D. simulans* (*w*Au in STC), and *w*Wil-transinfected *D. simulans* (*w*Wil in STC) lines. DNA is stained with DAPI (blue). Scale bar, 10 μm.

Quantification of the RI for infection of neuroblasts and whole larval CNS in *w*Wil/STC (Fig. 6B and C) confirmed the systemic nature of *w*Wil localization in *D. simulans* with no difference from native *w*Au in *D. simulans* (*P = *0.93 for neuroblasts and *P = *0.52 for larval brains, one-way ANOVA with Tukey HSD test), contrary to highly restricted tropism of *w*Wil in its native *D. willistoni* background (*P < *0.001, one-way ANOVA with Tukey HSD test). We found that the germ line of all three combinations was systemically infected with *Wolbachia*. Qualitative visual analysis revealed that transinfected *Wolbachia* titer was similar to that of its donor (*D. willistoni*) and not the recipient (*D. simulans*), which points to the key role of the microbe in titer regulation. However, we need to thoroughly quantify the bacterial load in egg chambers to test this hypothesis in our future research project. Interestingly, the infection of follicle cells in the adult ovaries of transinfected *w*Wil/STC flies was found to have a medium RI (Fig. 6D) compared to systemic *w*Au in *D. simulans* (*P < *0.001, one-way ANOVA with Tukey HSD test) and the highly restricted *w*Wil strain in *D. willistoni* (*P* < 0.001, one-way ANOVA with Tukey HSD test). Sequential FISH with *Wolbachia*-specific probes and immunofluorescence using anti-GABARAP and anti-FK2 antibodies on early embryos showed, contrary to *w*Wil in *D. willistoni*, no physical interaction of native *w*Au and *de novo w*Wil with autophagosomes and the absence of ubiquitination in *D. simulans* hosts (Fig. 7A and B). This observation was confirmed by quantitative colocalization of *Wolbachia* and the antibody signal using JACoP plugin in Fiji (Fig. S6D and E).

In summary, we conclude that the host background plays a major role in regulating the distribution of the endosymbiont in its tissues.

## DISCUSSION

Understanding the host-symbiont interaction regarding tropism and density control in the *Wolbachia*-*Drosophila* model system is of great importance for deciphering the essence of interkingdom relationships and also could be applied to *Wolbachia*-mosquito and other symbiotic associations. In our study, we analyzed bacterium-host interactions with a focus on microbe tropism by comparative and quantitative FISH analyses in several neotropical *Drosophila* species belonging to the willistoni and saltans species groups. We found that, similar to *w*Pau in *D. paulistorum*, native *w*Wil *Wolbachia* are locally restricted in larval and adult brains, whereas *D. tropicalis*, a close relative to *D. willistoni*, exhibits clear patterns of the SIT, similar to *w*Mel in D. melanogaster. In *D. septentriosaltans*, a representative of the saltans species group, we found no signs of tropism in host flies carrying the *w*Spt *Wolbachia* strain that also belongs to the *w*Au-like group (18, 31). In *D. sturtevanti*, however, *w*Stv *Wolbachia* are locally restricted, similar to the RIT of *w*Pau and *w*Wil in native willistoni group hosts. Interestingly, the characteristic restriction pattern of *w*Stv is also conserved in the closely related and newly described species *D. lehrmanae* (32) that carries a similar *w*Stv-like *Wolbachia* strain (W. J. Miller, unpublished data).

### Tissue tropism of *Wolbachia* has evolved at least twice in neotropical *Drosophila* hosts.

In the current study, we uncovered RIT patterns of the endosymbiont in three neotropical *Drosophila* hosts belonging to two different species groups that carry either *w*Au- or *w*Stv-like *Wolbachia* variants. This finding suggests that the local restriction of the endosymbiont evolved at least two times independently in neotropical *Drosophila* by targeting two different *Wolbachia* variants, the closely related and more ancestral *w*Au-like strain in the lineage of *D. paulistorum* and *D. willistoni* and the more recently acquired *w*Stv-like bacteria of *D. sturtevanti* and *D. lehrmanae* (32). As *w*Au-like *Wolbachia* are conspecific and the dominating, most likely ancestral, infection type of neotropical *Drosophila* species (18), we speculate that the last common ancestor of *D. sturtevanti* and *D. lehrmanae* carried a *w*Au-like strain too, which, in the following, got lost in competition with the arrival and successful establishment of the newly acquired *w*Stv-like strain. Under the assumption that the ancestral *w*Au infection was similarly restricted to defined tissues like *w*Wil and *w*Pau in their native willistoni group prior to *Wolbachia* strain replacement, we hypothesize that the newly arrived and possibly more aggressive *w*Stv variant became domesticated and attenuated in the same way as the ancestral *w*Au-like infection type before in WIL and PAU. As demonstrated by our transinfection experiments, it is most likely the host that mainly determines the tropism of the endosymbiont. By this, the host was already preadapted to costly *Wolbachia* infections by restricting and limiting the endosymbiont to defined germ line and somatic niches where the cost-benefit equilibrium was not disturbed. To test this hypothesis, however, more data on *Wolbachia* tropism will be essential from more species of the saltans group, since to date only systemic infections of *w*Au-like strains were found in *D. septentriosaltans* and *D. prosaltans* (Table 1 and Fig. S4C and D).

### *Wolbachia* tropism in adults is already determined in early embryos.

**FIG 8:**
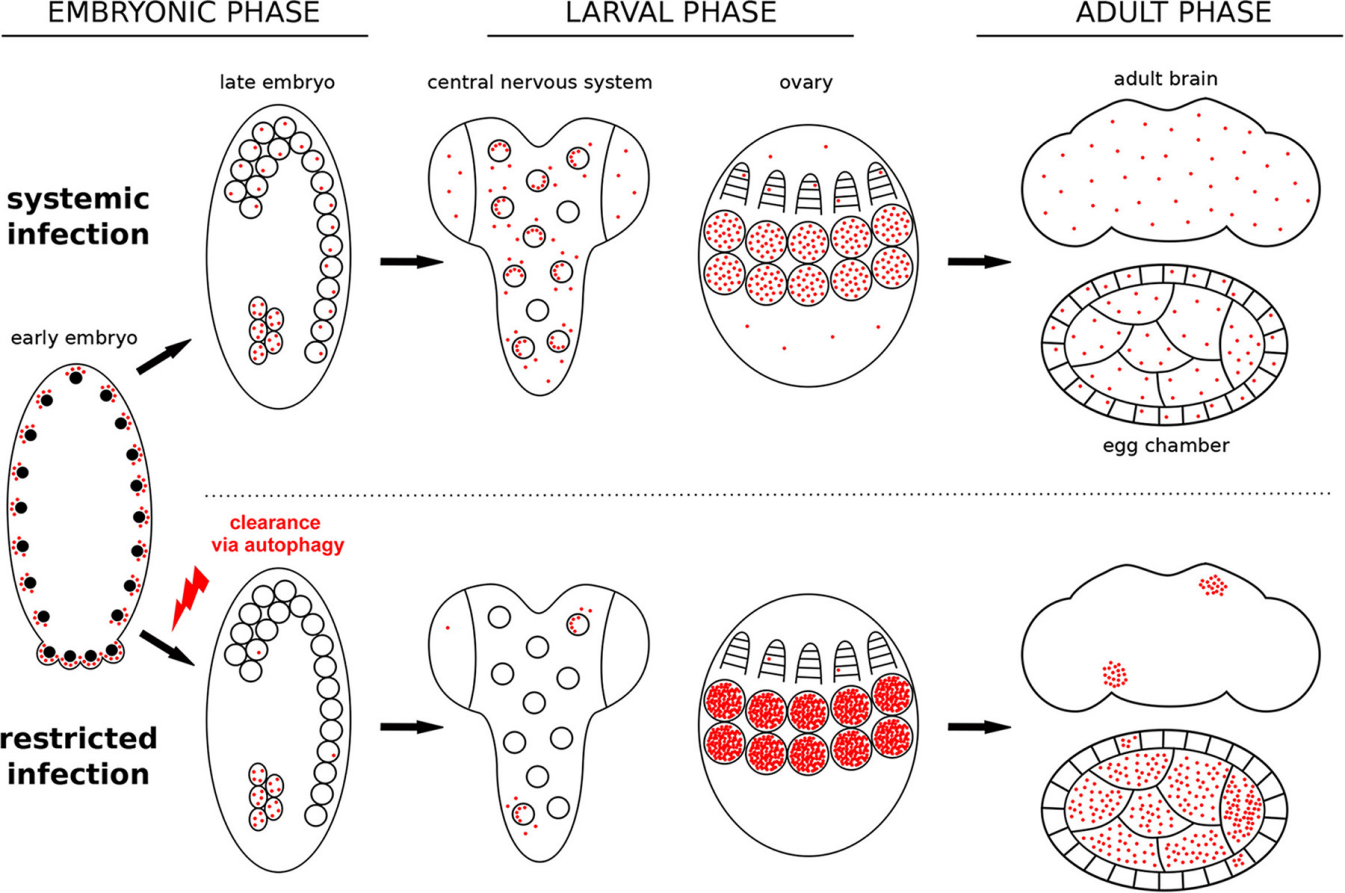
Schematic representation of *Wolbachia* distribution in systemic and restricting *Drosophila* species at different stages of host development (embryonic, 3rd-instar larval and adult brains and the female germ line). Active clearance of *Wolbachia* by autophagy occurs during early embryogenesis in RIT hosts, and the restricted pattern of infection is preserved at later stages. Note the higher infection density in germ line cells of 3rd-instar larvae and egg chambers of adult ovaries.

Our comparative studies performed by systematic *Wolbachia*-specific FISH uncovered that adult *D. paulistorum* and *D. willistoni* as well as *D. sturtevanti* flies, all natively infected by either *w*Au- or *w*Stv-like strains, share similar patterns of local symbiont restrictions in their respective brains and ovaries. This RIT tropism is already manifested in early-mid-embryogenesis by local restriction of the endosymbiont to the PGCs of the future germ line and a few cell clusters of the soma (including neuroblasts), suggesting that both stem cell types serve as the infection reservoir for the future imago (summarized in Fig. 8).

We hypothesize that the massive reduction of bacterial titer in early embryogenesis is necessary to alleviate the burden of infection for the adult fly establishing the cost-benefit equilibrium in the system, since systemically infected species of PAU, WIL, and STV were not observed in the lab or in recently collected wild specimens from French Guiana (data not shown). Analyses of bacterial densities during early embryogenesis demonstrated that all three neotropical *Drosophila* with RIT patterns exhibit high-titer *Wolbachia* infections (qualitatively summarized in Table 2). In *D. tropicalis*, a close relative of *D. paulistorum*, but exhibiting SIT, *Wolbachia* titer is stably low during the whole embryogenesis period.

**TABLE 2:**
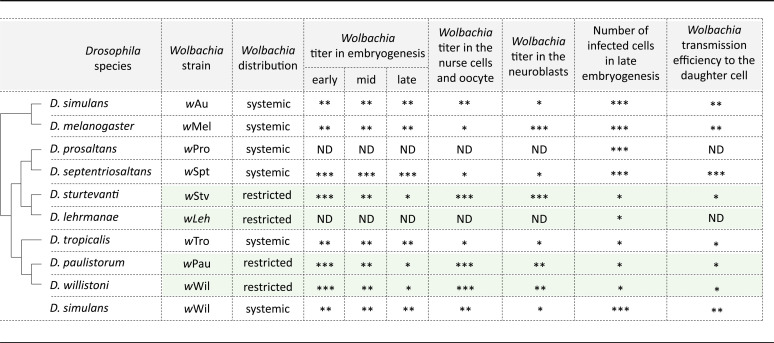
Summarized characteristics of *Wolbachia* strains in native and novel hosts analyzed in the present study^*a*^

a Asterisks indicate low (*), mid-range (**), and high (***) titer in the region of interest.

*Wolbachia* densities in embryos are strain specific and most likely determined by the number of bacteria transmitted into the unfertilized egg during oogenesis by posterior localization of the bacteria (12, 33). After fertilization during the early nuclear divisions, they presumably do not replicate but only segregate (34 and Miller, unpublished). Thus, it seems likely that the smaller numbers of *Wolbachia* observed in early-stage embryos of *D. tropicalis* are below a critical threshold and less costly in hosts with SIT. In RIT hosts, higher densities seem detrimental and, hence, are avoided by elimination from most somatic parts of the embryo, which, by natural selection, leads to endosymbiont’s restriction by the host. In contrast to *D. tropicalis*, in *D. septentriosaltans*, another species with systemic *Wolbachia* infection, the bacterial titer is stably high in embryogenesis; however, at later developmental stages and especially in the imago, the infection density decreases to MEL and TRO levels (Table 2). This reduction might occur due to a dilution effect via endosymbiont dissemination all over the developing organism during multiple cell divisions. In line with this idea, we previously demonstrated that some *D. paulistorum* semispecies harbor so-called low-titer *Wolbachia* infections (6) that are under the detection limit of standard PCR methods; hence, more sensitive methods are needed for their identification (6, 7, 19, 35, 36).

**FIG 9:**
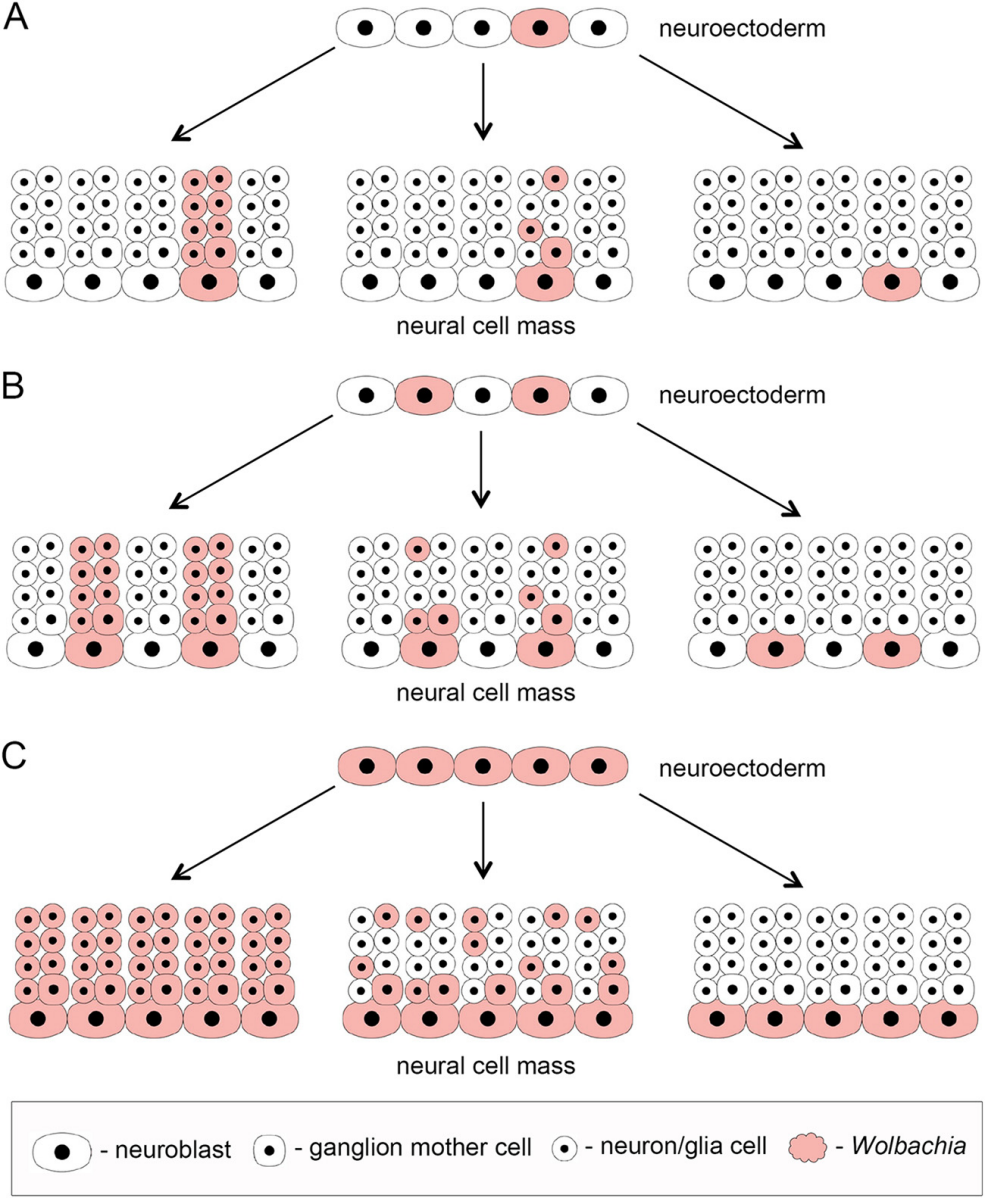
Description of all possible variants of *Wolbachia* distribution patterns during fly development exemplified on the central nervous system formation. The scheme demonstrates *Wolbachia* dissemination efficiency during mitosis of neuroblasts from the neuroectoderm with different starting numbers of infected stem cells (niches): low (A to C), moderate (D to F), and high (G to I). Each neural cell mass picture demonstrates the percentage of cells in the progeny of a single neuroblast receiving the infection.

We propose two main criteria for the establishment of *Wolbachia* tropism in symbiotic association: (i) the number of infected cells in late embryogenesis as a foundation of infection (Fig. 8) and (ii) the efficiency of *Wolbachia* transmission into dividing daughter cells during mitosis (Table 2). The first criterion represents a starting point with determined bacterial densities and localization, which is set in early-mid-embryogenesis. In RIT hosts, this is realized via directed elimination of bacteria from most somatic parts of the embryo and each infected pluripotent stem cell, like PGC or neuroblast, can be considered a niche for the endosymbiont (Fig. 8). The second criterion determines the future pattern of *Wolbachia* tropism in the adult fly by dissemination of infection from the niches by mitosis during development. The data on *Wolbachia* distribution in the nervous tissue of different *Drosophila* species across development demonstrated in this study and previously published (37, 38) support this idea (summarized in Fig. 9). In RIT hosts, the number of infected embryonic neuroblasts in the delaminated neuroectoderm is low due to extensive overall elimination of *Wolbachia* in the soma earlier in embryogenesis (Fig. 9A to C). Later in development, these restricted infection niches give rise to clusters of bacterial infection in the larval CNS and adult brains, which differ in size depending on the transmission efficiency (Fig. 9A to C). In the two systemic species with SIT, i.e., MEL and TRO, the ratio of infected neuroblasts is around 50% but the transmission efficiency is high enough to form multiple clusters of infection, generating the SIT pattern (Fig. 9D and E, respectively). In some species, not found so far, the dissemination of infection from the niches might be close to zero, occupying only neuroblasts (Fig. 9F and I). Finally, in SPT flies that also exhibit SIT, the number of infected neuroblasts is almost 100% and the efficiency of transmission is high, which leads to overall dissemination of infection in the adult fly (Fig. 9G and H).

Our *Wolbachia* transinfection experiment, bringing *w*Wil bacteria from the RIT host *D. willistoni* into the SIT background of *D. simulans*, demonstrated that it is mainly the host background that regulates the distribution pattern of infection in the soma. These data are not entirely consistent with previous results for different *Drosophila* tissues, where in most cases the *Wolbachia* strain determined the tropism (summarized in Table S1). Such a discrepancy might be explained by different *Wolbachia* strategies to infect reproductive and somatic tissues. For instance, our data demonstrated that *Wolbachia* localization pattern is not strictly regulated by the host in follicle cells of adult ovaries from the transinfected line (*w*Wil/STC).

10.1128/mbio.03863-21.9 TABLE S1 The role of bacterial and host factors in regulating the distribution and density of the infection in different *Drosophila* tissues demonstrated by cytological studies. Download Table S1, PDF file, 0.2 MB. Copyright © 2022 Strunov et al. 2022 Strunov et al. https://creativecommons.org/licenses/by/4.0/ This content is distributed under the terms of the Creative Commons Attribution 4.0 International license.

### Autophagy is a key mechanism, eliminating *Wolbachia* during early *Drosophila* embryogenesis.

In three out of six *Drosophila* species analyzed in the present study in detail, we observe high restriction of *Wolbachia* to certain areas in some somatic tissues and their accumulation in reproductive organs of the host. This restriction occurs in early embryogenesis during the narrow time window between cellularization (stage 5) and early gastrulation (stage 6 to 7), with the infection being substantially reduced in the soma but staying high in PGCs. This massive somatic elimination of *Wolbachia* coincides with maternal-to-zygotic transition in *Drosophila* embryogenesis, which is marked by extensive degradation of deposited maternal mRNA and activation of zygotic gene expression (39). In this study, we were able to dissect the process of *Wolbachia* clearance stepwise and demonstrated that bacteria are removed from the soma of RIT embryos via autophagy, which is schematically summarized in Fig. 8. To our knowledge, this is the first example of autophagy-mediated regulation of bacterial densities during early embryogenesis of the host.

**FIG 10:**
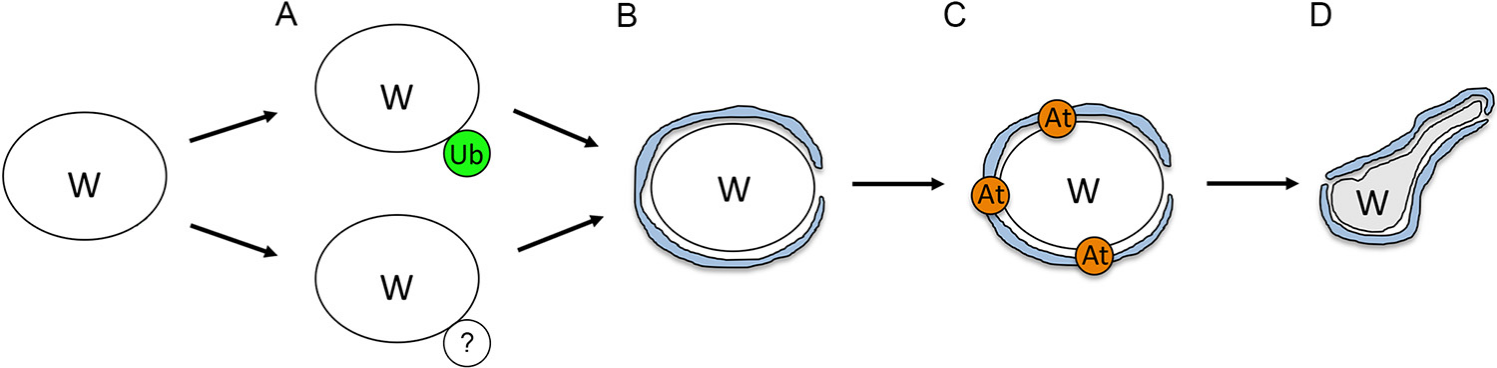
Scheme of *Wolbachia* elimination process during early host embryogenesis. (A) First step in infection elimination, ubiquitination (Ub), which is active in WIL hosts and absent in PAU and WIL. (B) Second step, the encircling of the bacteria by ER membranes. (C) Third step, the attraction of autophagy machinery to the vesicle formed by ER. (D) Last step, degradation of bacteria through an undescribed mechanism.

We propose that the first step of the bacterial elimination process is ubiquitination of the endosymbiont (Fig. 10A). It is generally used by cells to tag proteins for proteasomal degradation (40) but is also known for targeting intracellular bacteria for further elimination via autophagy during cellular defense against infections (41). In our study, however, we observe colocalization of ubiquitin with *Wolbachia* only in WIL species, whereas the other two RIT hosts, PAU and STV, showed low or no signs of it. Near absence of colocalization of ubiquitin with the native endosymbionts suggests that in these two hosts *Wolbachia* elimination occurs through a ubiquitin-independent pathway (42). In contrast to *w*Wil, *w*Pau and *w*Stv *Wolbachia* might have evolved a mechanism to remove the ubiquitination mark but still be cleared via autophagy through a different pathway. It was recently demonstrated that the *w*MelCS strain, but not the closely related *w*Mel, might have developed a trick to subvert the autophagy machinery by actively avoiding the ubiquitination in D. melanogaster hub cells (28).

The second step of bacterial elimination is characterized by ER membranes encircling the endosymbiont (Fig. 10B). Various intracellular bacteria exhibit intimate contacts with the ER, since it is a nutrient-rich organelle that is devoid of bactericidal effectors and thereby provides a safe niche for endosymbionts to survive and replicate (reviewed in reference 43). As demonstrated in earlier studies, *Wolbachia* exert close interactions with the ER membranes in different D. melanogaster tissues as well as in fly-derived cell lines (44–48). Additionally, endosymbionts most likely receive their third outer membrane from the ER, which helps them to escape from cellular defense systems (reviewed in reference 12). The ER, however, is not always a friendly environment for bacteria. Disruption of the secretory pathway by active endosymbiont interaction, causing ER stress, might lead to recognition by the innate immune system and cell defense response (reviewed in reference 43). Moreover, the ER seems to provide a cradle for autophagosome formation (49), which might ameliorate the elimination of bacteria.

In our TEM studies, we uncovered intimate interaction of rough ER membranes with *Wolbachia* in PAU embryos during the symbiont´s elimination process, which is in sharp contrast to MEL embryos with rare and significantly less intimate contacts. Based on the results of our antibody staining against GABARAP, we speculate that ER membranes surrounding *Wolbachia* in PAU embryos serve as a scaffold for autophagosome formation. The role of ER membranes in the degeneration of bacteriocytes was also demonstrated for the symbiotic Buchnera-Aphid system (50). Additionally, ER encircling was recently demonstrated for damaged mitochondrial elimination via mitophagy in mouse embryonic fibroblasts (51). Very similar to our observation, not fully functional mitochondria are first ubiquitinated and then surrounded by ER strands, which provide a platform for mitophagosome formation and further degradation of the organelle. Given that mitochondria have alphaproteobacterial ancestry, both observations mentioned above strongly support our hypothesis of ER playing a key role in the somatic elimination of the alphaproteobacterial *Wolbachia* in early RIT embryos by forming a cradle for autophagosome maturation.

The third step of the bacterial elimination process is attraction of the autophagy machinery followed by autophagosome maturation (Fig. 10C). It is known that autophagy plays an important role in defending the host cell against pathogens, but in some cases the autophagy machinery can be hijacked by the intruder for its own survival (reviewed in reference 52). In some systems autophagy might be a key player in maintaining the cost-benefit equilibrium (27, 28, 53).

In our RIT hosts, we observed *Wolbachia* accumulation mostly in PGCs during embryogenesis, whereas the rest of infection in the soma is massively eliminated and subsequently restricted to certain isolated areas. Eventually, adult flies exhibit highly abundant infection within the reproductive part of the gonad (nurse cells and oocyte) and restricted infection in somatic parts, like follicle cells and nervous tissues. The evolution of restricted tropisms of the endosymbiont to embryonic PGCs can be explained from the perspective of both symbiotic partners. On the one hand, for ensuring their own maternal transmission, *Wolbachia* might specifically avoid autophagy in gonad precursors by actively blocking it with unknown effector proteins, which are released via type IV secretion system (54). As shown in the literature, some bacteria can counteract the host defense system by selectively preventing any of these three steps: detection, autophagy initiation, or autophagosome formation (reviewed in references 55 and 56). This defense strategy of the symbiont also coincides with the downregulation of autophagy genes as observed in ovaries of the wasp *Asobara tabida* and the woodlouse *Armadillidium vulgare* (57, 58). Additionally, a recent study demonstrated that *w*MelCS strain of *Wolbachia* evolved a mechanism to subvert host autophagy in order to survive in hub cells, and both *w*Mel and *w*MelCS can avoid elimination in the developing egg (28).

On the other hand, the PGCs themselves might lack extensive autophagic activity and thereby provide a safe environment for the *Wolbachia* to survive, replicate, and be successfully transmitted via oocytes. In contrast to somatic cells, PGCs are transcriptionally quiescent during early embryonic stages (59) and activated only at later stages during their migration (60). It is conceivable that autophagy is blocked or impeded in germ line stem cells during this quiescent state. Although, for this study, we did not conduct additional experiments to decipher the mechanism of preservation of bacterial infection in PGCs, it appears to be more plausible that the cell specificity in development is a key regulator for *Wolbachia’*s fate. Therefore, during this critical step in early embryogenesis, PGCs are serving as a safe haven for the maternally transmitted endosymbiont within the hostile somatic environment of massive autophagy in *Drosophila* species with the RIT phenotype.

Another interesting question is why species with systemic infection do not clear *Wolbachia* from their cells during embryogenesis. The bacteria might be able to hide from elimination by the host because of unique surface markers that cannot be recognized by the autophagy machinery. Alternatively, *Wolbachia* can subvert the cell machinery and use it for their needs, as was recently described for *w*MelCS strain (28).

The final step of the bacterial elimination process is degradation (Fig. 10D). In our TEM studies, we observed several abnormalities of *Wolbachia* morphology in the soma of PAU embryos during elimination of infection, like stretching, bending, and membrane vesiculation. Usually dying *Wolbachia* exhibit shriveled, electron-dense structures surrounded by autophagosomal membranes (2, 46, 61, 62), but the abnormalities observed in our study on RIT embryos are unique and represent an uncommon way of bacterial degradation.

Although observed for the organelles and not yet for bacteria, similarly stretched and bent structures were reported for stressed mitochondria in murine embryonic fibroblasts (63) and other mouse tissues (64), linking these morphological deformations to autophagosome maturation by engulfing the cytoplasm and subsequent organelle degradation. In the latter more recent study, actual autophagosome formation was not confirmed by antibody staining, but the authors speculated that mitochondria can undergo a self-destruction process called mitoautophagy (64). Morphologically similar ultrastructural abnormalities were also found with plastids of *Brassica napus* plants during the developmental switch from microspores to embryogenesis. Here, the authors experimentally verified these abnormal plastids with autophagosome formation and further elimination (65). Taken together, our discovery of similar deformities of *Wolbachia* morphology in embryogenesis of RIT *Drosophila* hosts most likely represents the first report of a noncanonical degradation process of bacteria through autophagy that was only found in organelles before.

### Conclusions.

In the present study, we reconstructed the mechanism of restricting *Wolbachia* infection by autophagy in three different neotropical *Drosophila* species. These data present a unique way of symbiont density regulation by the host during a specific period in embryogenesis, which coincides with maternal-to-zygote transition. They also demonstrate how the cost-benefit equilibrium between the host and the symbiont is further maintained over host development by eliminating the microbe from most of the soma of the embryo to reduce potential future costs but keeping a safe niche in the reproductive part for the transmission for the symbiont. It is still unclear how *Wolbachia* escapes elimination in PGCs and in the soma of systemic species. One possibility is a unique marker on the bacterial surface, which is specifically recognized by a native host, but further transinfection experiments with various *Wolbachia* strains into different *Drosophila* backgrounds might give us the answers.

## MATERIALS AND METHODS

### Fly stocks and husbandry.

Seven different species from four *Drosophila* subgroups were used in this study: D. melanogaster (MEL), *D. simulans* (melanogaster subgroup), *D. paulistorum* (PAU), *D. willistoni* (WIL), *D. tropicalis* (TRO) (willistoni subgroup), *D. septentriosaltans* (SPT) (saltans subgroup), and *D. sturtevanti* (STV) (sturtevanti subgoup). All the species mentioned above were naturally infected with specific *Wolbachia* strains (*w*Mel, *w*Au, *w*Pau, *w*Wil, *w*Tro, *w*Spt, and *w*Stv, respectively). Additionally, the stably transinfected *w*Wil/STC line was used in the experiment, generated in 2006 by injecting *w*Wil *Wolbachia* from *D. willistoni* into *D. simulans* STC early embryos, which were cleared from the native *w*Au *Wolbachia* with antibiotics. For more details on flies used in the study, see Table 1. All lines were kept at 22 to 25°C on a 12-h light-dark cycle and fed a typical molasses, yeasts, cornmeal, and agar diet.

### RNA-DNA fluorescent *in situ* hybridization.

Tissues (adult brains, larval CNS, adult ovaries, larval ovaries, and hemocytes) from at least 10 females per *Drosophila* species/line were dissected in ice-cold RNase-free 1× phosphate-buffered saline (PBS), fixed in 3.7% formaldehyde in RNase-free PBS for 15 to 20 min at room temperature, and consequently washed 3 times, 5 min each time, with PBTX (1× PBS, 0.3% Triton X-100). Embryos from listed *Drosophila* species were collected and fixed according to a standard protocol (66).

All fixed samples were hydrated in prewarmed 4× SSC (1× SSC is 0.15 M NaCl plus 0.015 M sodium citrate) buffer with 10% formamide and hybridized at 37°C overnight in the same buffer containing 10% dextran sulfate and 0.5 nmol W1/W2 probes specifically targeting *Wolbachia* 16S rRNA (67) labeled with Oregon Green (488) or Texas Red (596) fluorophore. Samples were then washed twice for 30 min at 37°C in prewarmed 4× SSC buffer with 10% formamide. For preparation of larval CNS and ovaries and adult ovaries, tissues were additionally incubated in Alexa Fluor 488 phalloidin (1:100 dilution in 1× PBS; Invitrogen, USA) for 1 h at room temperature to stain F-actin. Finally, after washing samples 2 times with 1× PBS, they were mounted in Roti-Mount FluorCare with 4′,6-diamidin-2-phenylindol (DAPI) (Carl Roth, Germany) on microscope slides.

Samples were analyzed on Olympus FluoView FV3000 confocal microscope. Beam paths were adjusted to excitation/emission peaks of used fluorophores: 569/591 nm for CAL Fluor Red 590 (*Wolbachia*), 488 nm for phalloidin, and 350/450 nm for DAPI.

### FISH combined with immunofluorescence (FISH/IF).

For combination of FISH with antibody staining, we first conducted *in situ* hybridization as described in the section above. After washing steps in prewarmed 4× SSC buffer, samples were incubated in 5% bovine serum albumin (BSA) for 1 h at room temperature with constant shaking. They were washed once with 1% BSA and incubated with a primary antibody (diluted in 1× PBTX with 1% BSA) overnight at 4°C constantly shaking. The following day the samples were washed 3 times, 10 min each time, in 1× PBTX and incubated in a secondary antibody (diluted in 1× PBTX with 1% BSA) for 1 h at room temperature with constant shaking. After washing 3 times, 10 min each time, with 1× PBTX, samples were stained with Alexa Fluor 488 phalloidin (1:100 dilution in 1× PBS; Invitrogen, USA). They were then washed 2 times with 1× PBS and mounted in Roti-Mount FluorCare with DAPI (Carl Roth, Germany) on microscope slides.

### Antibodies.

The following primary antibodies were used in this study: anti-Deadpan (guinea pig, polyclonal; 1:1,000 [68]), anti-Asense (guinea pig, polyclonal; 1:100) (68), anti-Repo (rabbit, polyclonal; 1:1000; gift of G. Technau), anti-Vasa (rat, polyclonal; 1:500; gift of A. Ephrussi), anti-GABARAP (rabbit, polyclonal; 1:200; E1J4E, monoclonal antibody number 13733; Cell Signaling Technologies; gift of S. Martens), anti-FK2 (mouse, monoclonal; 1:200; gift of F. Ikeda), and anti-GRP78/BiP (rabbit, polyclonal; 1:500; Abcam, Cambridge, UK). The following secondary antibodies were used in this study: goat anti-mouse Alexa Fluor 488 (1:500), goat anti-mouse Cy5 (1:500), goat anti-rabbit Alexa Fluor 488 (1:500), goat anti-guinea pig Cy3 (1:500), and goat anti-rat Alexa Fluor 488 (1:500). All secondary antibodies were obtained from Invitrogen USA.

### Transmission electron microscopy.

*Drosophila* embryos were collected the same way as for FISH and then fixed in 2.5% (wt/vol) glutaraldehyde in 0.1 M sodium cacodylate buffer (pH 7.2) for 2.5 h. This was followed by three washes in the same buffer for 5 min each and postfixation in 1% (wt/vol) OsO_4_ and 0.8% (wt/vol) potassium ferrocyanide for 1 h. Samples were then placed in a 1% aqueous solution of uranyl acetate (Serva, Heidelberg, Germany) for 12 h at 4°C and dehydrated in an ethanol series (30%, 50%, 70%, and 96% for 10 min and 100% for 20 min) and acetone (twice for 20 min). Ultrathin sections of embedded samples (Agar 100 resin; Agar Scientific Ltd., Essex, UK) were obtained with a Reichert-Jung ultracut microtome, stained with Reynolds lead citrate, and examined in an FEI Tecnai 20 electron microscope (FEI Eindhoven, Netherlands) equipped with a 4K Eagle charge-coupled device camera. Images were processed with Adobe Photoshop.

### Analysis and quantification of *Wolbachia* localization in the tissue.

We define a restriction index (RI) to quantify the pattern of *Wolbachia* localization as number of uninfected cells divided by total number of cells:

$$\text{RI}\;=\;\frac{F_{\text{uninfected}}}{F_{\text{total}}}$$

$F_{\text{uninfected}}$ and $F_{\text{total}}$ in adult brains and larval CNS were calculated by superimposing a grid (25 by  25 μm) on the whole tissue image in Photoshop CS6 and quantifying the number of uninfected and total number of grids containing the tissue. The RI value varied from 0 (no restriction) to 1 (full restriction). In total, 10 samples per *Drosophila* species and each tissue were analyzed (more than 1,200 grid cells for adult brains and approximately 400 grid cells for larval nervous tissues of each species).

The RI of infection in adult and larval ovaries was calculated by dividing the number of uninfected follicle cells from a central section of egg chamber (for the former) or somatic cells related to terminal filament (for the latter) to the total number of cells analyzed. In total, 10 samples per *Drosophila* species and each tissue were analyzed (more than 400 cells for adult ovaries and more than 170 cells for larval ovaries of each species). The RI of infection in somatic cells around primordial germ cells (PGCs) in embryos was quantified by drawing a 50- by 50-μm square around PGCs, counting the number of uninfected cells within this square and dividing it by the total number of cells. In total, 10 samples per *Drosophila* species and tissue were analyzed (more than 300 cells for each species).

The RI of infection in neuroblasts of embryonic head was quantified by counting the number of uninfected cells (stained with anti-Deadpan antibody specific to neuroblasts) and dividing it by the total number of neuroblasts. In total, 10 samples per each *Drosophila* species and each tissue were analyzed (more than 400 neuroblasts for each species).

Aggregation of *Wolbachia* in larval CNS was calculated by quantifying the average number of infected neighboring cells forming a cluster in each tissue. In total, 8 samples per each *Drosophila* species were analyzed (61 to 65 cell clusters for SIT, 26 to 32 cell clusters for RIT, and 56 cell clusters for the transinfected line).

*Wolbachia* density within a neuroblast of larval CNS and within an egg chamber of an ovary or an embryo was quantified with Fiji (30) by measuring the area of bacterial signal within the region of interest (ROI) and dividing it by the total area of the ROI. In total, at least 5 to 10 samples per *Drosophila* species and tissue were analyzed. The detailed description of this procedure can be found in reference 20.

### Statistics.

All statistical analyses were carried out using R version 3.3.2 (R-Core Team, 2020). For *Wolbachia* distribution in adult and larval brains and ovaries, we analyzed the count data based on generalized linear models (GLM) with a Poisson error structure. To test for significance of a given predictor variable, we compared the full model, including all factors, to a reduced model excluding the given factor by analysis of deviance with χ^2^ tests using the R function *anova*. For the rest of the data, we assume that the data are normally distributed and calculated one-way ANOVAs. We further applied *post hoc* Tukey HSD test to test for significant difference among factor levels using the R function *TukeyHSD*.
