# Supplementary figures and images for: Restriction of Wolbachia Bacteria in Early Embryogenesis of Neotropical Drosophila Species via Endoplasmic Reticulum-Mediated Autophagy
Source: mBio. 2022 Mar 31;13(2):e03863-21. doi: 10.1128/mbio.03863-21 (PMC9040723; doi:10.1128/mbio.03863-21)

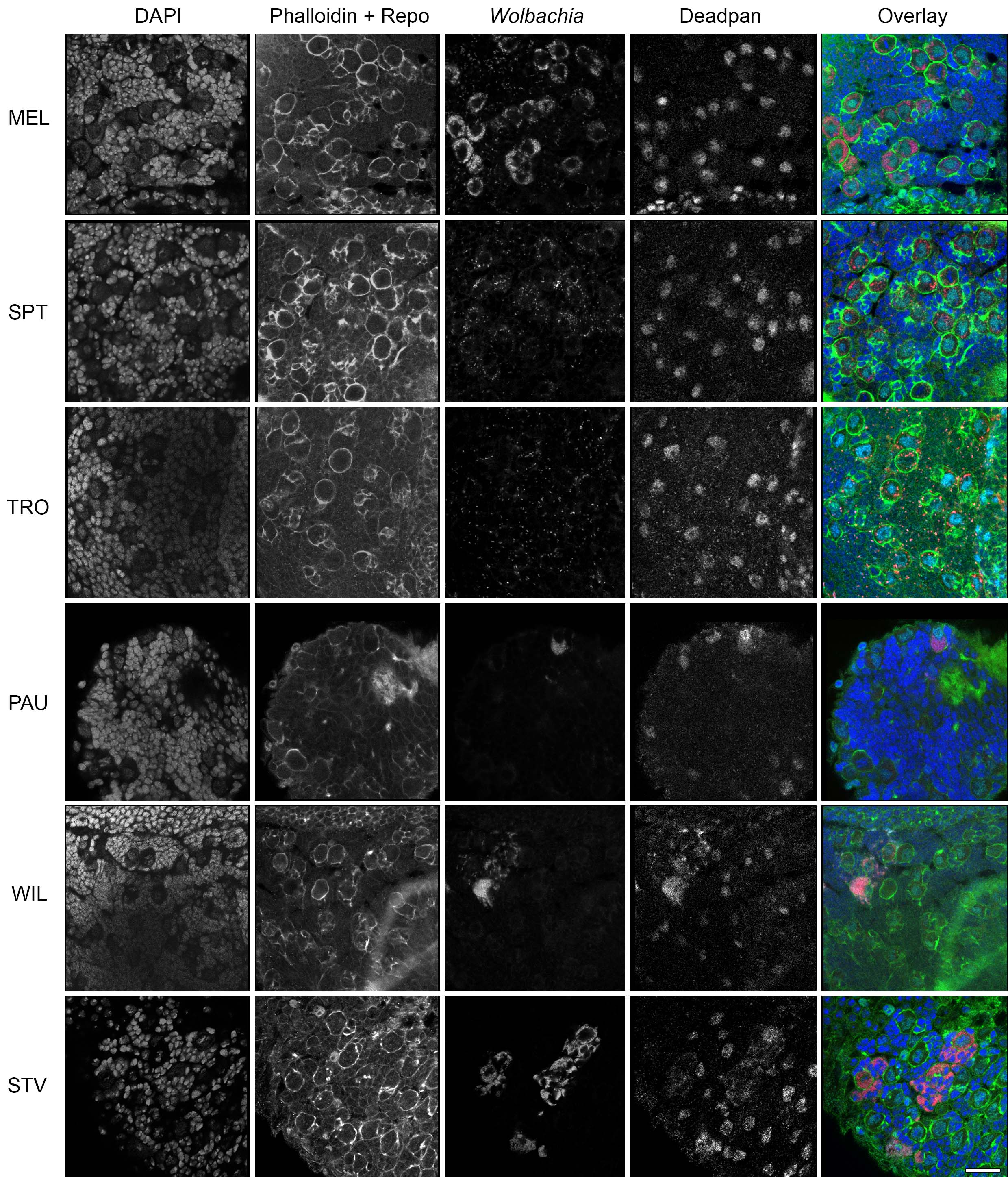

Supplement: FIG S1 [file mbio.03863-21-sf001.jpg]

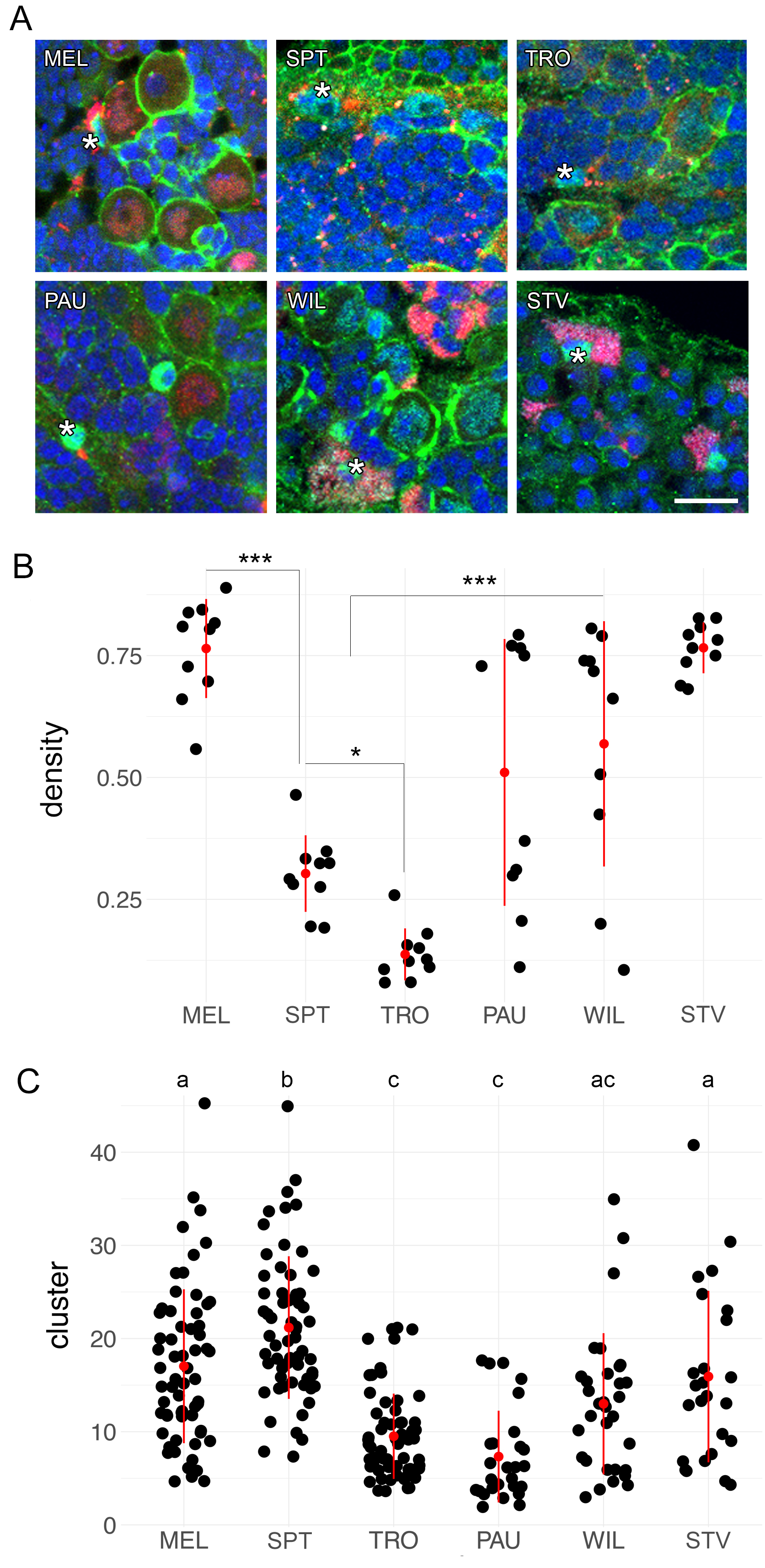

Supplement: FIG S2 [file mbio.03863-21-sf002.jpg]

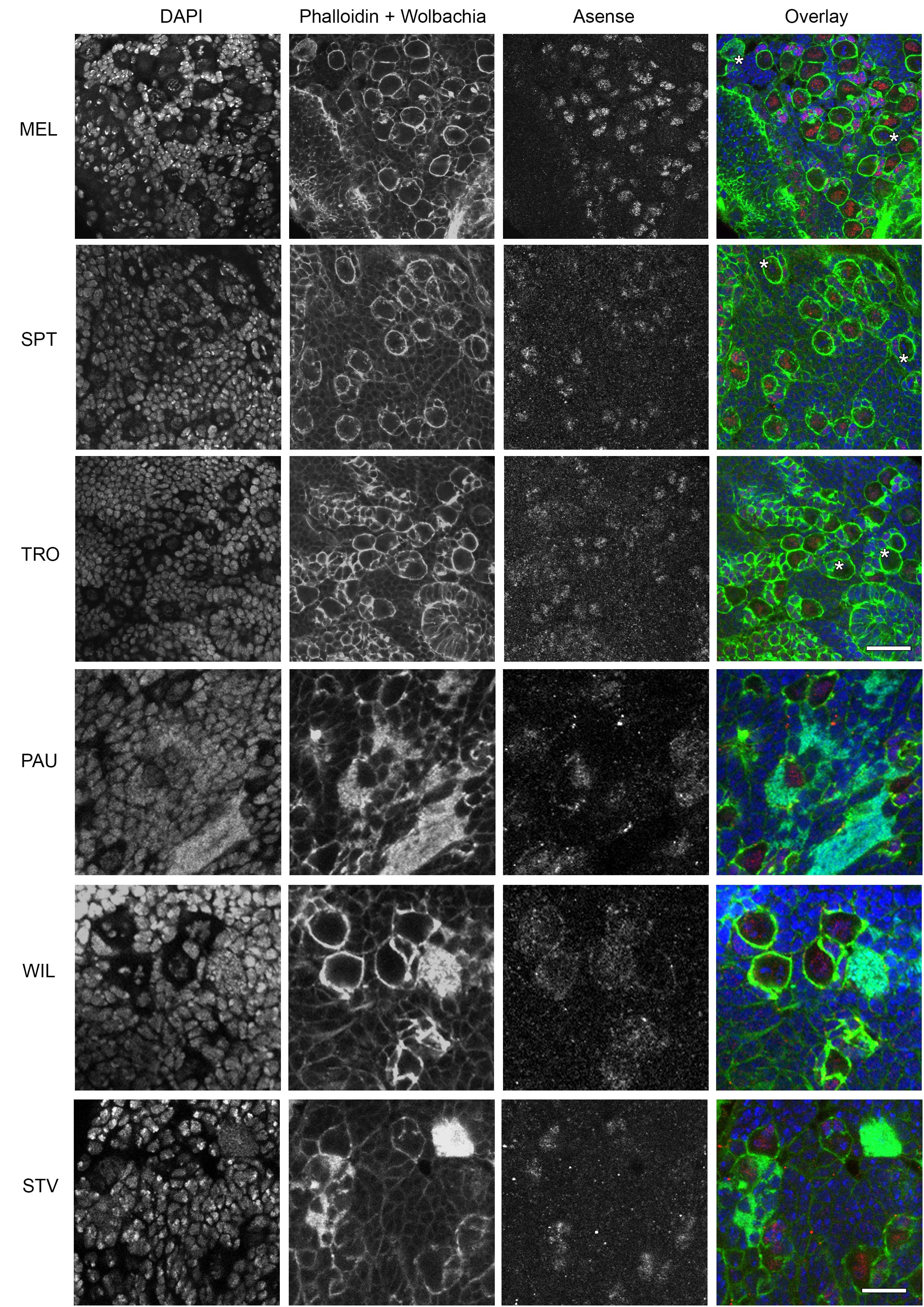

Supplement: FIG S3 [file mbio.03863-21-sf003.jpg]

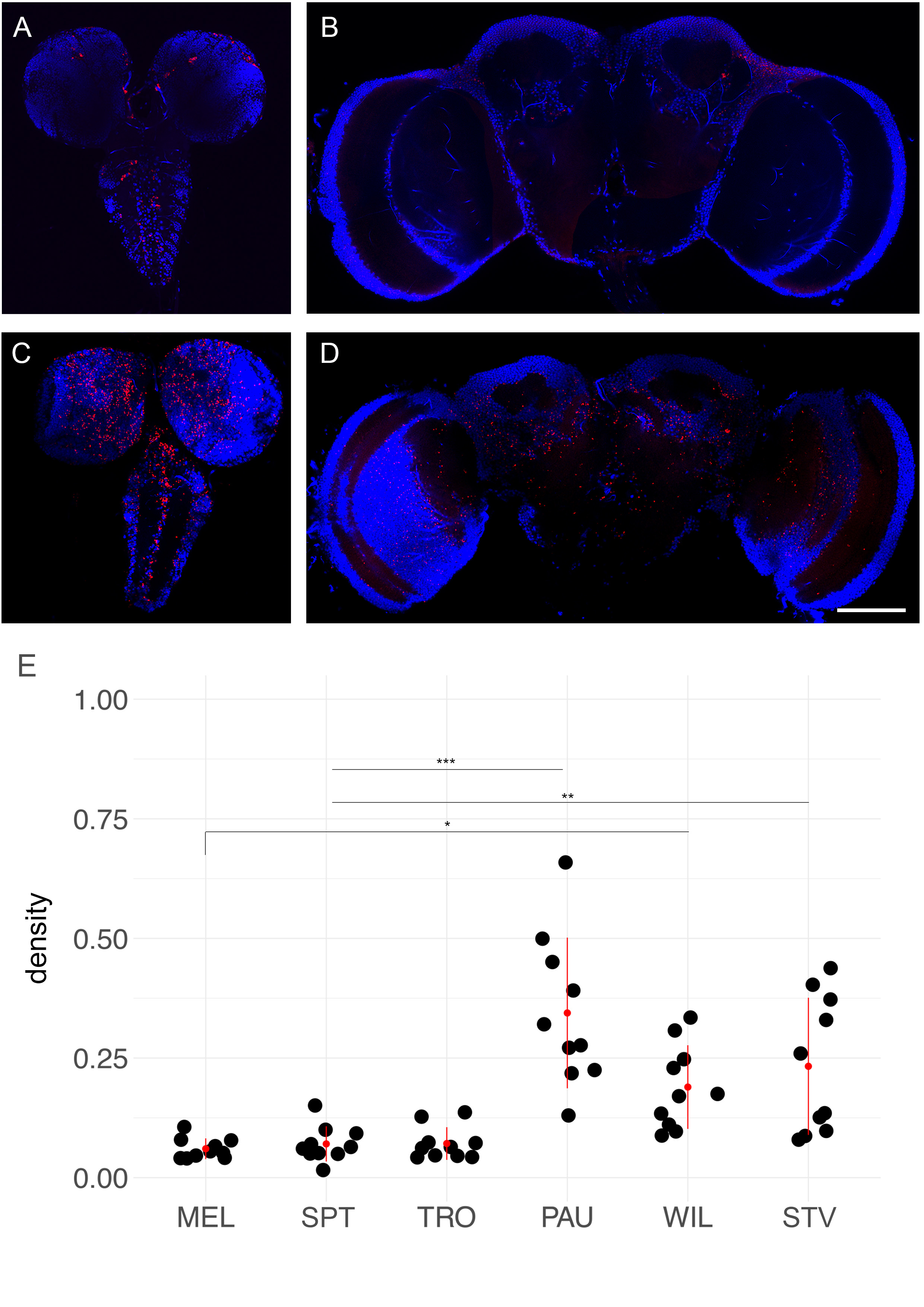

Supplement: FIG S4 [file mbio.03863-21-sf004.jpg]

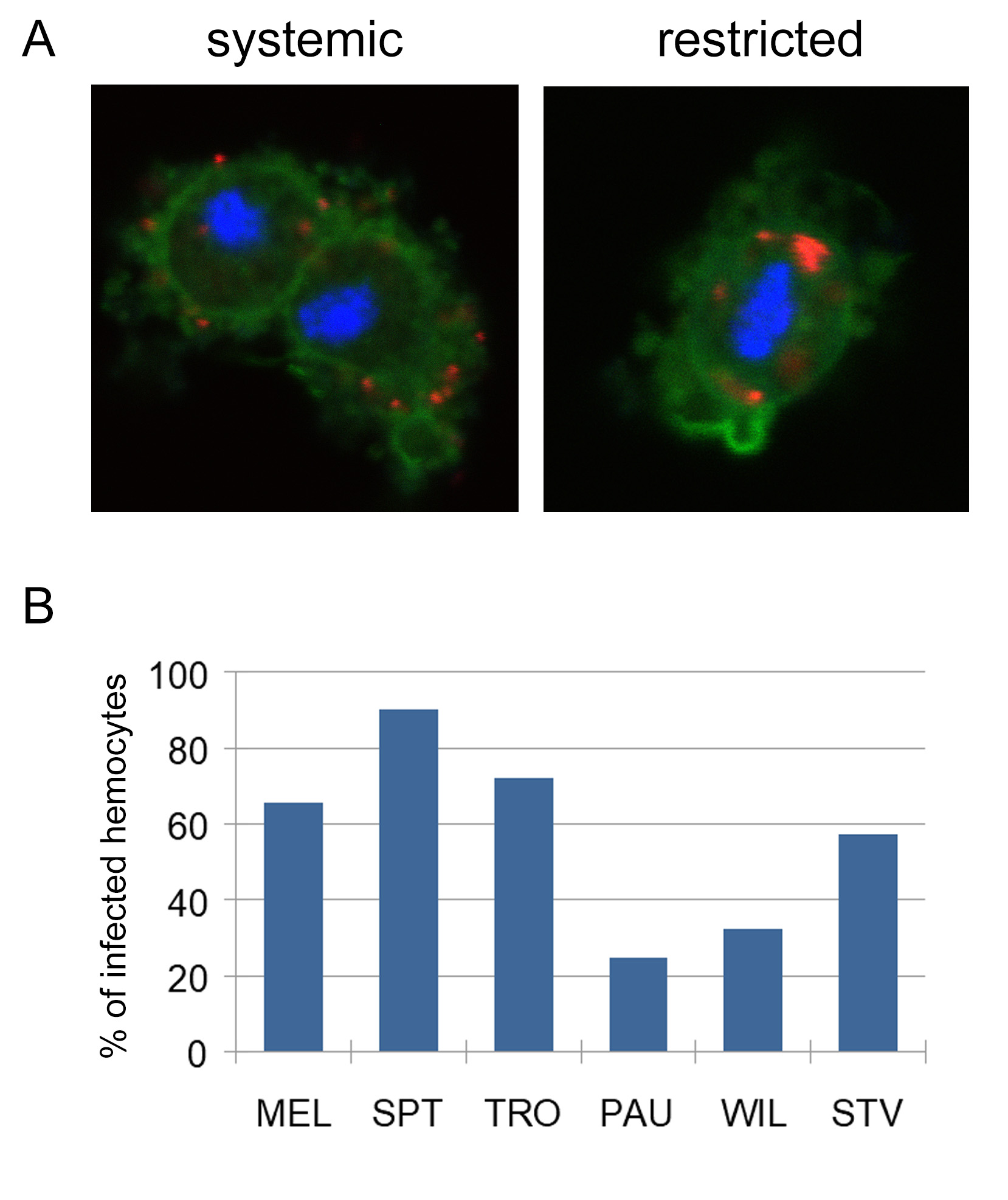

Supplement: FIG S5 [file mbio.03863-21-sf005.jpg]

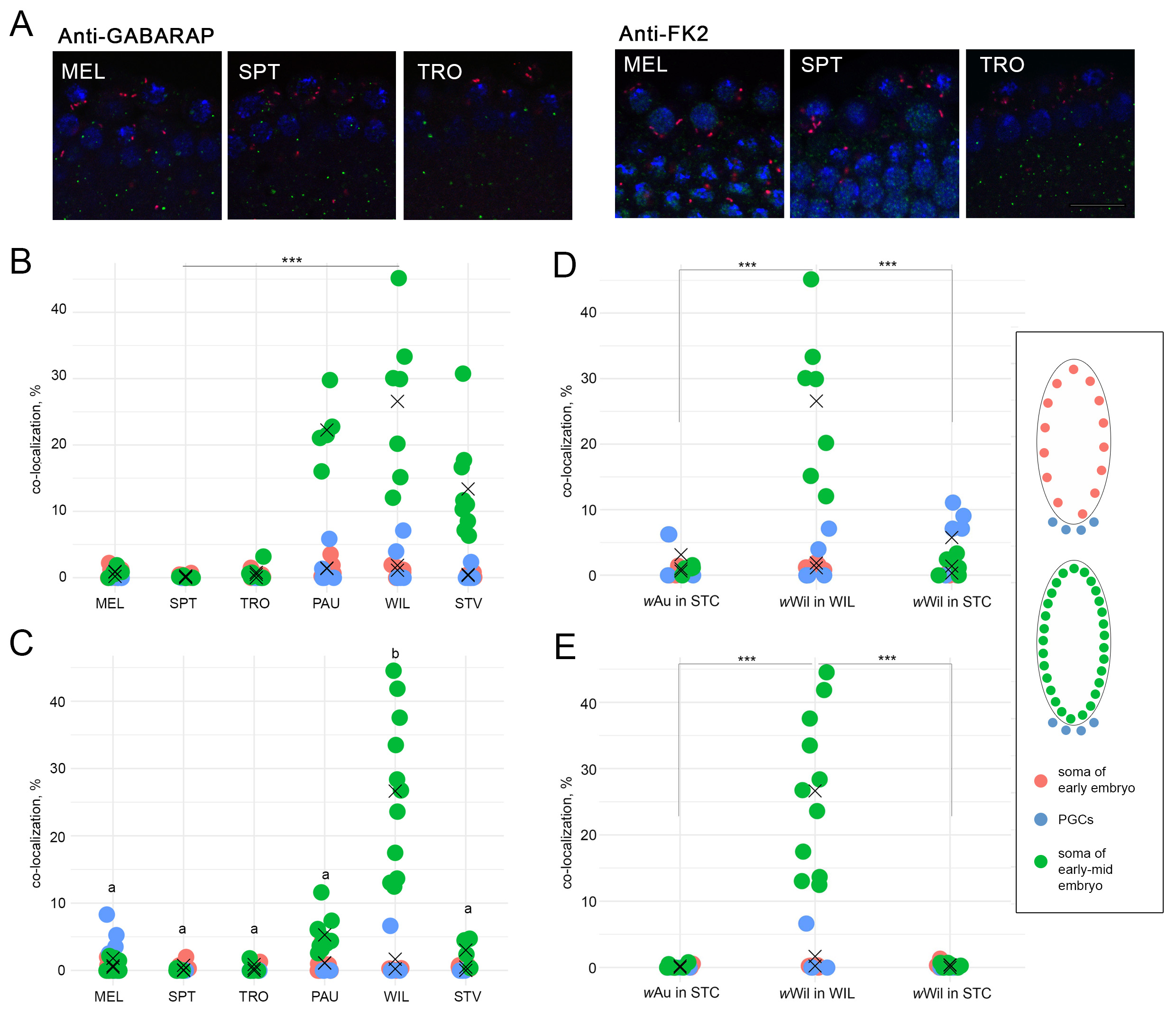

Supplement: FIG S6 [file mbio.03863-21-sf006.jpg]

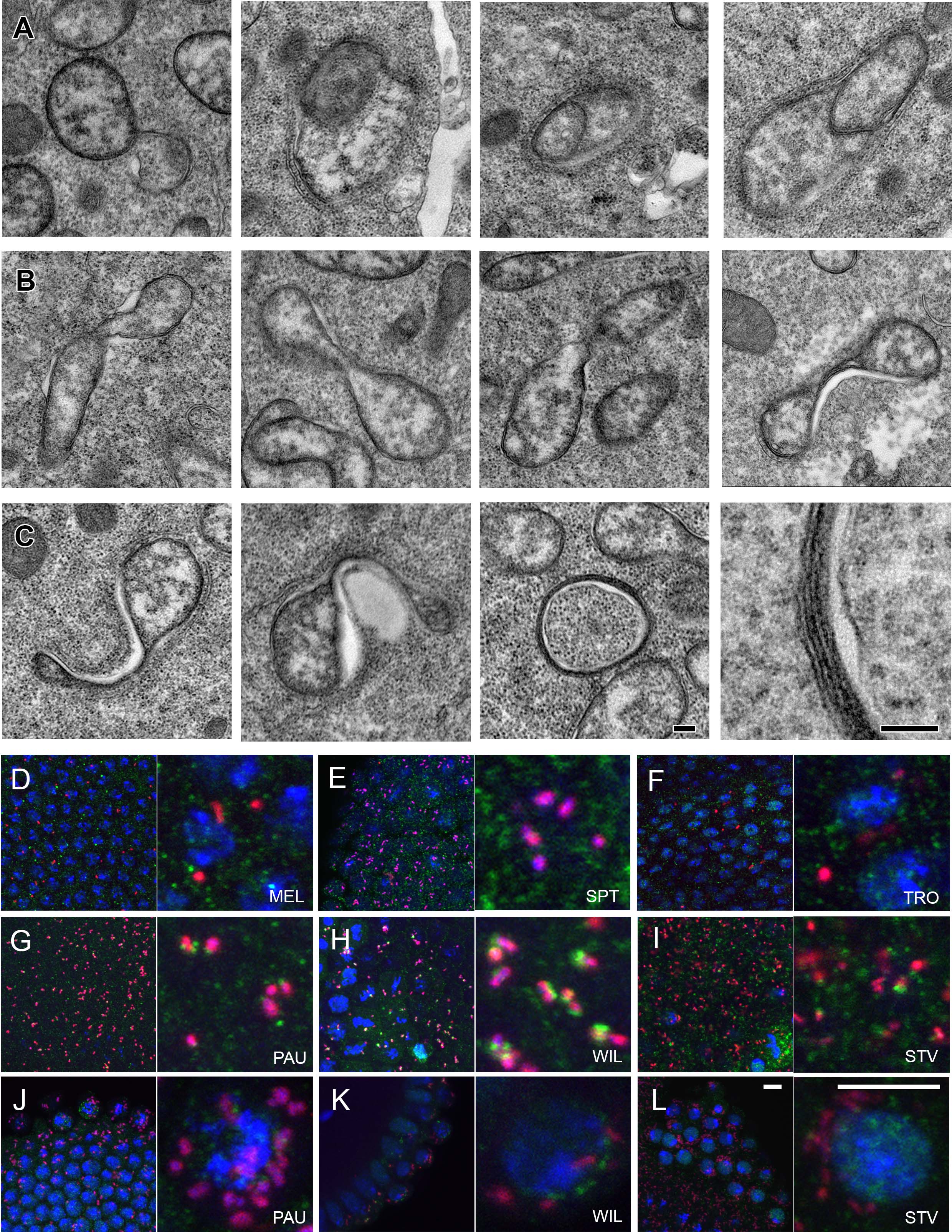

Supplement: FIG S7 [file mbio.03863-21-sf007.jpg]

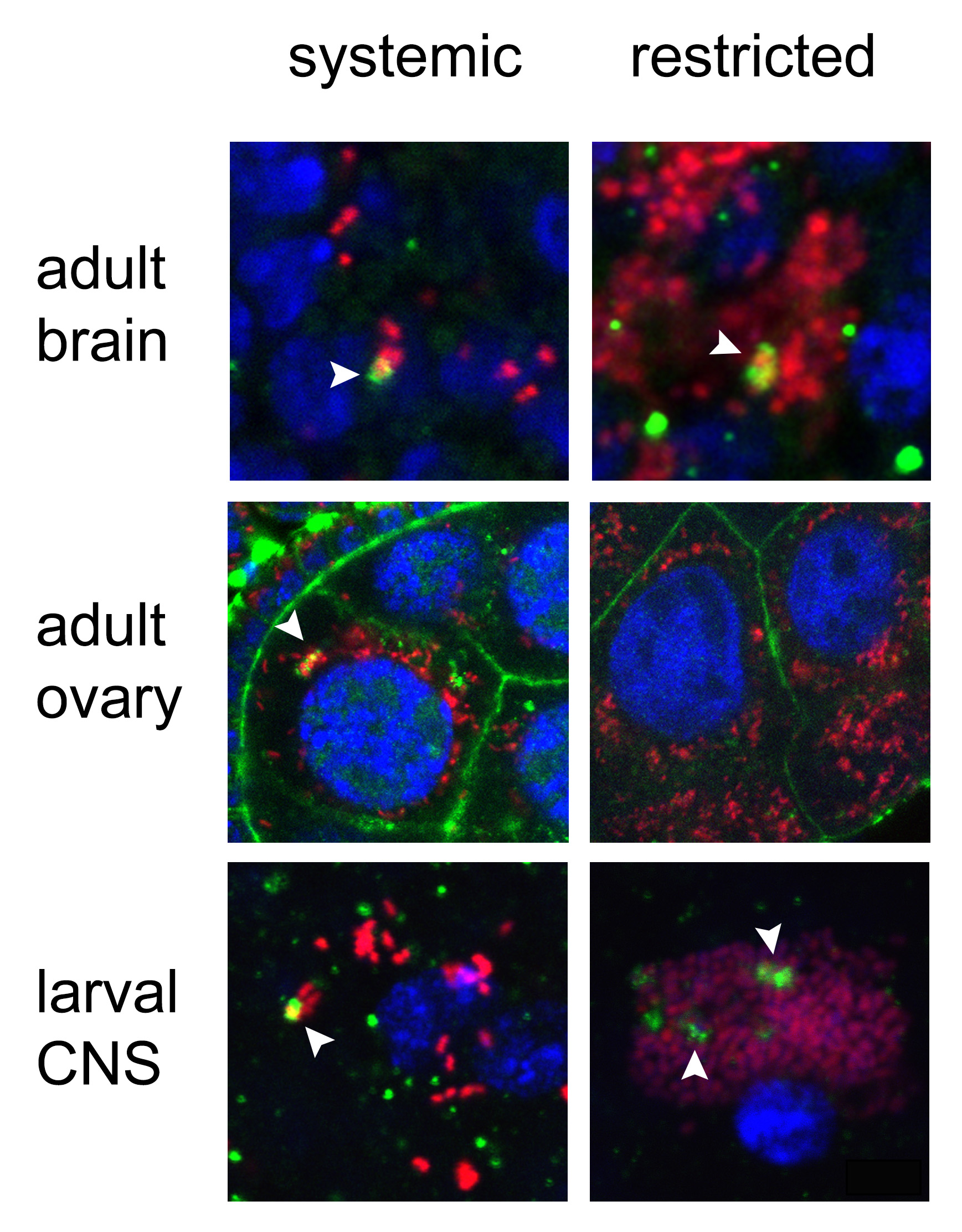

Supplement: FIG S8 [file mbio.03863-21-sf008.jpg]
