# Supplementary material for: Restriction of Wolbachia Bacteria in Early Embryogenesis of Neotropical Drosophila Species via Endoplasmic Reticulum-Mediated Autophagy
Source: mBio. 2022 Mar 31;13(2):e03863-21. doi: 10.1128/mbio.03863-21 (PMC9040723; doi:10.1128/mbio.03863-21)
Supplement: TABLE S1 [file mbio.03863-21-st001.pdf]

references	tissue	tropism regulated by	titer regulated by
Veneti et al., 2004	ovaries and testes	*Wolbachia*	host
Serbus and Sullivan, 2007	ovaries	*Wolbachia*	host
Albertson et al., 2013	CNS	*Wolbachia*	*Wolbachia* + host
Toomey et al., 2013	ovaries	*Wolbachia*	NA
Toomey, Frydman, 2014	testes	*Wolbachia* + host	NA
this study	CNS + ovaries	host	*Wolbachia*

1) Serbus, L.R., Sullivan, W., 2007. A cellular basis for Wolbachia recruitment to the host germline. PLoS Pathog 3, e190. https://-doi.org/10.1371/journal.ppat.0030190

2) Toomey, M.E., Panaram, K., Fast, E.M., Beatty, C., Frydman, H.M., 2013. Evolutionarily conserved Wolbachia-encoded factors control pattern of stem-cell niche tropism in Drosophila ovaries and favor infection. Proc Natl Acad Sci U S A 110, 10788–10793. https://doi.org/10.1073/pnas.1301524110

3) Toomey, M.E., Frydman, H.M., 2014. Extreme divergence of Wolbachia tropism for the stem-cell-niche in the Drosophila testis. PLoS Pathog 10, e1004577. https://doi.org/10.1371/journal.ppat.1004577
